# The coupling of transcriptome and proteome adaptation during development and heat stress response of tomato pollen

**DOI:** 10.1186/s12864-018-4824-5

**Published:** 2018-06-08

**Authors:** Mario Keller, SPOT-ITN Consortium, Kamila Lucia Bokszczanin, Hamed Bostan, Arnoud Bovy, Palak Chaturvedi, Yuanyuan Chen, Maria Luisa Chiusano, Nurit Firon, Sotirios Fragkostefanakis, Rina Iannacone, Sridharan Jegadeesan, Hanjing Li, Celestina Mariani, Dominik Marko, Anida Mesihovic, Florian Müller, Puneet Paul, Marine Paupiere, Ivo Rieu, Klaus Dieter Scharf, Enrico Schleiff, Wolfram Weckwerth, Peter Winter, Wim Vriezen, Stefan Simm

**Affiliations:** 1 Department of Biosciences, Molecular Cell Biology of Plants, Goethe University, D-60438 Frankfurt am Main, Germany; 2 Solanaceae Pollen Thermotolerance Initial Training Network Consortium, Frankfurt am Main, D-60438 Germany; 3 Frankfurt Institute of Advanced Studies, D-60438 Frankfurt am Main, Germany

**Keywords:** Transcriptomics, Proteomics, Pollen development, Heat stress

## Abstract

**Background:**

Pollen development is central for plant reproduction and is assisted by changes of the transcriptome and proteome. At the same time, pollen development and viability is largely sensitive to stress, particularly to elevated temperatures. The transcriptomic and proteomic changes during pollen development and of different stages in response to elevated temperature was targeted to define the underlying molecular principles.

**Results:**

The analysis of the transcriptome and proteome of *Solanum lycopersicum* pollen at tetrad, post-meiotic and mature stage before and after heat stress yielded a decline of the transcriptome but an increase of the proteome size throughout pollen development. Comparison of the transcriptome and proteome led to the discovery of two modes defined as direct and delayed translation. Here, genes of distinct functional processes are under the control of direct and delayed translation. The response of pollen to elevated temperature occurs rather at proteome, but not as drastic at the transcriptome level. Heat shock proteins, proteasome subunits, ribosomal proteins and eukaryotic initiation factors are most affected. On the example of heat shock proteins we demonstrate a decoupling of transcript and protein levels as well as a distinct regulation between the developmental stages.

**Conclusions:**

The transcriptome and proteome of developing pollen undergo drastic changes in composition and quantity. Changes at the proteome level are a result of two modes assigned as direct and delayed translation. The response of pollen to elevated temperature is mainly regulated at the proteome level, whereby proteins related to synthesis and degradation of proteins are most responsive and might play a central role in the heat stress response of pollen.

**Electronic supplementary material:**

The online version of this article (10.1186/s12864-018-4824-5) contains supplementary material, which is available to authorized users.

## Background

Pollen, the male gametophyte, has served as model system for analyzing cell growth and development in a variety of plant species including *Arabidopsis thaliana* [1–3] and *Oryza sativa* [4, 5]. The development of pollen can be divided into two phases, namely microsporogenesis and microgametogenesis. First, the diploid pollen mother cell, also known as microsporocyte, undergoes meiotic division to give rise to a tetrad of four haploid microspores. After release of the post-meiotic microspores from the tetrad, each of them undergoes an asymmetric mitotic division to form a mature bicellular pollen [6, 7]. The analysis of the developmental stages of pollen has mainly been conducted at the transcriptome level. To date, for *A. thaliana* it is estimated that up to 7235 genes are expressed in mature pollen, while in earlier developmental stages like uninucleate microspores the number of expressed genes is with 11,565 far higher [8]. Genes expressed in mature pollen were shown to be enriched for specific processes including signaling, cell wall metabolism and cytoskeleton but underrepresented for energy metabolism, transport, transcription and translation [8]. In addition to transcriptome analyses, there is also a need for proteomic studies to obtain a more complete picture about active processes and their regulation at transcriptome and proteome level. In the last decade extensive proteomic studies within the Solanaceae family and in particular in tomato (*Solanum lycopersicum*) have been conducted [9]. One of these studies was focused on the proteomic profiling of five pollen developmental stages of tomato, namely microsporocyte, tetrads, microspores, polarized microspores and mature pollen [10]. The authors were able to identify 345 proteins in the diploid microsporocyte, whereas in the four haploid stages a higher number of proteins was detected. The number of detected proteins increased from 655 proteins in tetrads to 1337 proteins in the polarized microspore followed by a slight decrease to 1104 proteins in mature pollen. Further, proteins with increased levels in specific developmental stages were identified and linked to processes required at a certain time during pollen development. Multi-omics studies that analyzed transcriptomic and proteomic data in pollen highlighted differences between both levels [11–14]. For example the proteome content of mature pollen is enriched for proteins associated with carbohydrate and energy metabolism, which was not apparent in the transcriptome [11].

Next to differences in transcriptome and proteome content, the developmental stages differ in their sensitivity to abiotic stresses [15]. In particular, heat stress (HS) has a strong impact and may lead to reduced pollen viability and germination [16]. The highest sensitivity of pollen occurs between meiosis and the first mitosis, whereas later developmental stages are more tolerant to high temperature [17]. At the molecular level, HS leads to denaturation and aggregation of proteins, which impairs important functional processes [18]. To maintain protein homeostasis during HS, specific heat shock proteins (Hsps) are synthesized and accumulate in the cytosol and organelles. Hsps are classified according to their molecular weight into seven families: Hsp100, Hsp90, Hsp70, Hsp60, Hsp40, small Hsp (sHsp) and Hsp10 [19–21]. In plants the different Hsp families comprise multiple members, whereby some are heat-inducible and others constitutively expressed [22]. Expression of Hsps is mainly controlled by HS transcription factors (Hsfs), which induce transcription of HS-inducible genes by binding of heat shock elements located upstream of the transcription start site [23, 24]. An accumulation of Hsps upon HS was also described for early developmental stages of pollen, whereas later stages like mature or germinating pollen lack most of the general HS induced Hsps [17, 25–28]. Further, it was shown that Hsps also accumulate in early developmental stages of non-stressed pollen, indicating a developmental priming to protect stages ranging from microsporocyte to early microspores against abiotic stresses [10, 29, 30]. Beside the importance of Hsps it could be shown that a complex network of HS related proteins of different functions is involved to keep up the homeostasis of the cell [19].

The aim of this study was to characterize the pollen proteome along the course of development as well as the analysis of protein level changes in developing pollen under elevated temperature. We set a focus on the proteome as it provides direct information about enzymatic and regulatory activity. Further, we extended our analysis by transcriptomic data for a better understanding of the relationship between transcriptome and proteome. In detail, we performed a combination of high throughput transcriptome and proteome profiling for three developmental pollen stages of tomato under HS and control (CO) conditions. Comparison of transcriptome and proteome levels enabled the identification of two translational modes active in developing pollen. One leads to a direct translation of transcripts, whereas the other one includes the storage of transcripts until translation in a later stage (delayed translation). Both modes show a prioritization of certain cellular processes. Further, we were able to show that under HS a high number of proteins undergo drastic changes in their abundance, whereas in the transcriptome only a small number of transcripts was affected. A final analysis of Hsp families revealed strong differences in their regulation at the transcriptome and proteome level as well as between developmental stages.

## Results

### Transcriptome and proteome analysis in developmental stages of pollen under CO and HS

Male gametophyte development is characterized by massive transcriptome and proteome changes required for the successful generation of mature haploid pollen from diploid microsporocytes. High temperatures affect both transcriptome and proteome landscape. To get a detailed picture of such alterations, we analyzed the transcriptome and proteome of pollen from different developmental stages from tomato plants (cv. Red Setter) either kept at 25 °C as control or exposed to 1 h at 38 °C and then allowed to recover for 1.5 h at 25 °C. Pollen was isolated at tetrad, post-meiotic and mature stage. Red Setter is considered as HS sensitive cultivar regarding pollen thermotolerance [31]. Further, Red Setter has been used for the generation of a TILLING population, allowing the selection of mutants for interesting candidate genes from transcriptomic and proteomic analyses [32]. The chosen treatment temperature is within the optimum temperature range (37 to 40 °C) to induce the tomato HS response such as accumulation of Hsps [33].

We constructed transcriptome libraries based on the massive analysis of cDNA ends (MACE; [34]) technique and proteome libraries based on liquid chromatography tandem-mass spectrometry (LC-MS/MS). Read counts obtained from the MACE libraries were normalized for differing sequencing depth leading to the measure of transcripts per million (TPM). To ensure that a gene is actually detected at the transcriptome level, we defined a threshold based on transcripts of 16 genes encoding for proteins of the light-harvesting complex. This method was recently established for transcriptomic data of pollen and is based on the assumption that these transcripts are not supposed to be detected due to the absence of chloroplasts in pollen [35]. We observed a maximal TPM value of 4.3 for these transcripts and consequently set a threshold of 5 TPM for a transcript to be considered as detected (Additional file 1: Figure S1). To define the protein abundance, the mass spectra obtained from the LC-MS/MS were quantified using label-free quantification (LFQ) intensities via the MaxQuant software package [36], whereby some of the proteins were grouped (protein groups).

The transcriptome decreases from tetrads to mature pollen (Table 1; Additional file 2: Table S1). While in tetrads around 11,000 transcripts were detected, the number of detectable transcripts in post-meiotic and mature pollen decreased by up to 50% (~ 9000 post-meiotic; ~ 5000 mature). For the proteome we observed an opposite effect. Here, we determined around 700 protein groups in mature pollen, while the number declined to ~ 550 in post-meiotic pollen and ~ 400 protein groups in tetrads.

**Table 1:** Number of detected transcripts and protein groups in MACE and LC-MS/MS libraries, respectively

	MACE		LC-MS/MS	
Stage	CO	HS	CO	HS
Tetrad	11,168	11,125	343	450
Post-meiotic	8678	9403	564	560
Mature	5559	5293	722	709

Transcripts and protein groups were marked as detected if they were identified in at least two biological replicates

In total, 12,606 and 12,684 different transcripts were detected across all developmental stages under CO and HS, respectively (Additional file 1: Figure S2A and B). Under CO 4538 (36.0%) and under HS 4332 (34.2%) are common to all stages. (Additional file 1: Figure S2A and B). The total number of detected protein groups across all stages was 957 in CO and 1062 in HS samples, whereby the fraction of common protein groups between the stages was much lower (~ 20%) than the fraction of common transcripts (~ 35%) (Additional file 1: Figure S2C and D).

**Fig. 1:**
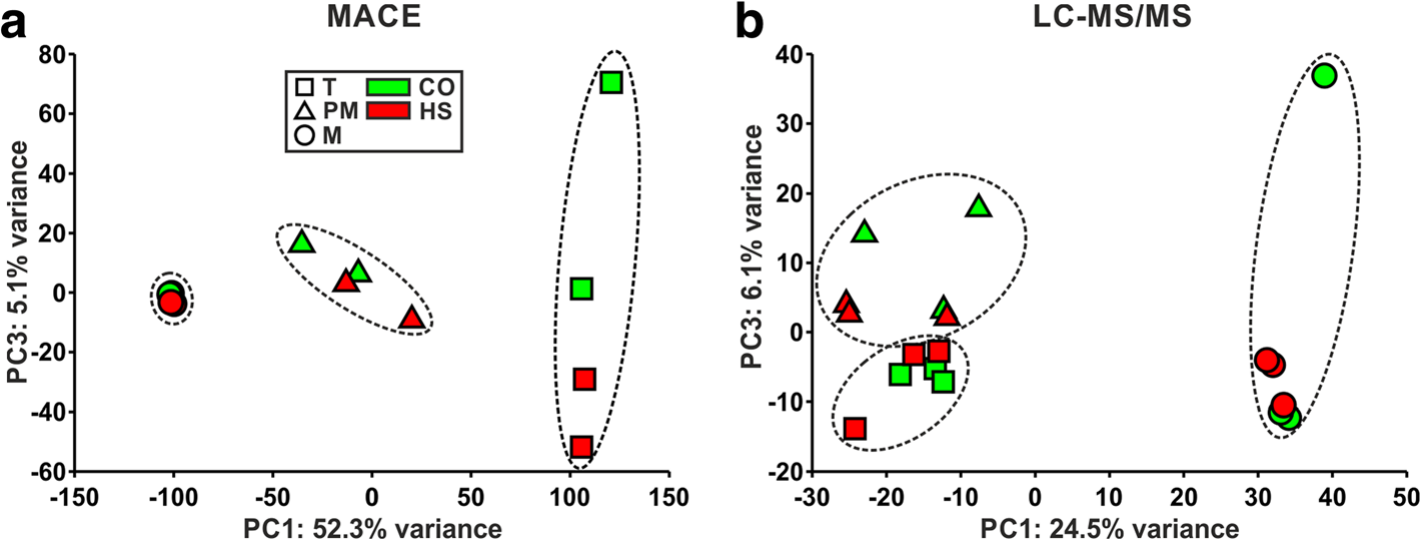
PCA plots of pollen developmental stages under CO and HS. **a** and **b** PCA plots for tomato pollen at tetrad (square), post-meiotic (triangle) and mature stage (circle) under CO (green) and HS (red) based on transcript (**a**, MACE) and protein group levels (**b**, LC-MS/MS). Axes are labeled with the variance explained by the first and third dimension, respectively

A principal component analysis (PCA) of the MACE and LC-MS/MS libraries revealed a separation of the developmental stages (Fig. 1). For the MACE libraries principal component 1 (PC1), which accounts for 52.3% of the variance inherent in the data, was already sufficient to separate the developmental stages (Fig. 1a). PC3 revealed a slight separation of CO and HS libraries for tetrads, whereas a clear separation of CO and HS was not apparent for post-meiotic and mature pollen (Fig. 1a). For the LC-MS/MS libraries PC1 accounts for only 24.5% of the variance and only separated mature pollen from the other two developmental stages. Here, PC3 includes the information separating tetrads and post-meiotic pollen (Fig. 1b), but not CO and HS conditions. Interestingly, inclusion of PC2 revealed no better separation of the developmental stages for the analysis of both, transcriptome and proteome levels (Additional file 1: Figure S3).

### Direct and stage-delayed translation in pollen

We targeted both, the identification of similarities and differences between transcriptome and proteome content within a single developmental stage as well as between the three different developmental stages. For this purpose, we performed k-means clustering (k = 15) for CO samples on the basis of the relative transcript levels (transcript cluster 1–15; Tr1–15) and the relative protein group levels across the three developmental stages (protein cluster 1–15; Pr1–15; Additional file 3: Table S2). Proteins of the same protein group were subsequently treated indepently.

First, we identified developmental stage-increased transcript (Tr1 – Tr6) and protein (Pr1 – Pr6) clusters. Clusters were defined as stage-increased if the respective stage covered two-thirds (0.66) of the relative abundance in the cluster profile. By this procedure we ensured that the transcripts of these clusters had, for the respective stage, at least two times increased transcript or protein levels in comparison to the other two stages. After filtering the content of these clusters for genes detected at both, the transcriptome and proteome level, we identified 229 tetrad (Tr1 and Tr2), 109 post-meiotic (Tr3 and Tr4) and 58 mature (Tr5 and Tr6) increased transcripts as well as 25 tetrad (Pr1), 141 post-meiotic (Pr2 and Pr3) and 520 mature (Pr4, Pr5 and Pr6) increased proteins (Additional file 3: Table S2 highlighted in bold).

**Fig. 2:**
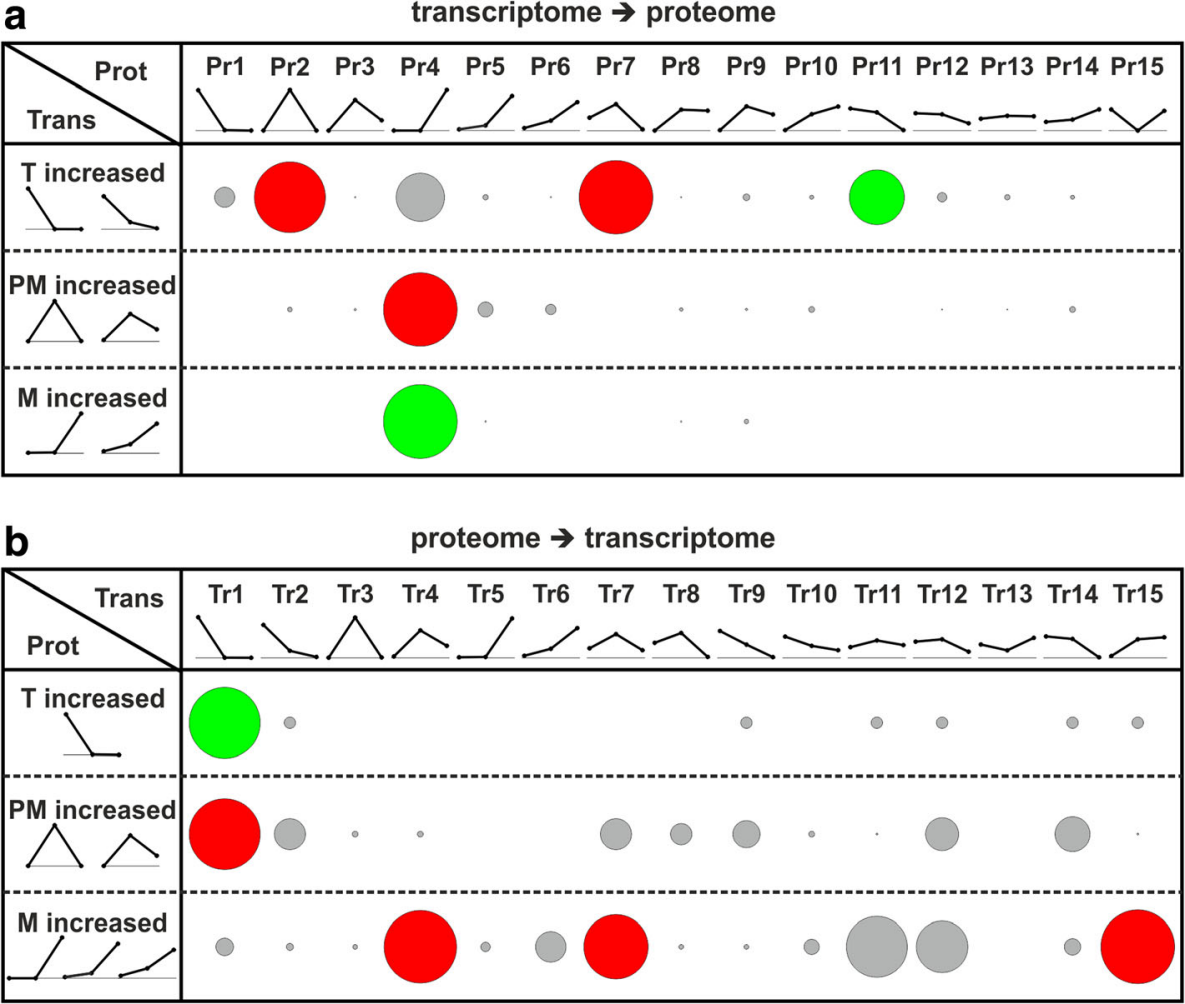
Identification of genes showing direct and delayed translation. **a** Genes showing stage-increased transcript levels in tetrads (T increased), post-meiotic (PM increased) and mature pollen (M increased) were traced in the protein clusters. **b** Genes showing stage-increased protein levels (T increased, PM increased and M increased) were traced in transcript clusters. Circle sizes are proportional to the gene overlap between T, PM and M increased and protein (**a**) or transcript clusters (**b**). For this purpose, the gene overlaps were scaled between 0 (lowest gene overlap) and 1 (highest gene overlap) independently for T, PM and M increased. Further, we defined gene overlaps as showing direct (green circles) or delayed translation (red circles) if they had a scaled value above 0.5. Direct translation was present when increased transcript levels were accompanied by increased protein levels, whereas delayed translation was apparent when the increase of protein levels was postponed by one developmental stage

Next, we calculated an overlap matrix, which indicated the number of shared genes between transcript and protein cluster pairs (Additional file 1: Figure S4). Subsequently, we focused on the overlap of genes between the stage-increased transcript clusters and each protein cluster (Fig. 2a circles) as well as between the stage-increased protein clusters and each transcript cluster (Fig. 2b circles). Here, we observed two translational modes. The first implies a ‘direct translation’ (Fig. 2 green circles), which is present when increased transcript levels are accompanied by increased protein levels in the same developmental stage (e.g. overlap T increased with Pr11; Fig. 2a). The second represents a ‘delayed translation’ (Fig. 2 red circles), as the increase of protein levels is postponed by one developmental stage with respect to the increase of transcript levels (e.g. overlap T increased with Pr2; Fig. 2a). Consequently, we divided the stage-increased genes into these two sets with respect to their translational mode for each developmental stage.

We observed 54 genes showing direct translation in tetrads, which had tetrad increased transcripts (T increased) and proteins in cluster Pr11 (Fig. 2a) or tetrad increased proteins (T increased) and transcripts in cluster Tr1 (Fig. 2b). Further, we identified 108 genes with delayed translation in tetrads, which had tetrad increased transcripts (T increased) and proteins in cluster Pr2 or Pr7 (Fig. 2a) or post-meiotic increased proteins (PM increased) and transcripts in cluster Tr1 (Fig. 2b). For post-meiotic pollen we identified no sufficient number of genes with direct translation but 223 genes with delayed translation. These genes had post-meiotic increased transcripts (PM increased) and proteins in cluster Pr4 (Fig. 2a) or mature increased proteins (M increased) and transcripts in cluster Tr4, Tr7 or Tr15 (Fig. 2b). For mature pollen we identified 53 genes with direct translation, which had mature increased transcripts (M increased) and proteins in cluster Pr4 (Fig. 2a). Genes with delayed translation in mature pollen are not extractable from our experiment as the mature stage was the last pollen stage inspected. So far, we could show the existence of two translational modes in developing pollen. Of the genes with stage-increased transcript or protein levels 53 and 54 were shown to be directly translated in tetrads and mature pollen, respectively. Further, 108 and 223 genes show delayed translation in tetrads and post-meiotic pollen, respectively.

**Fig. 3:**
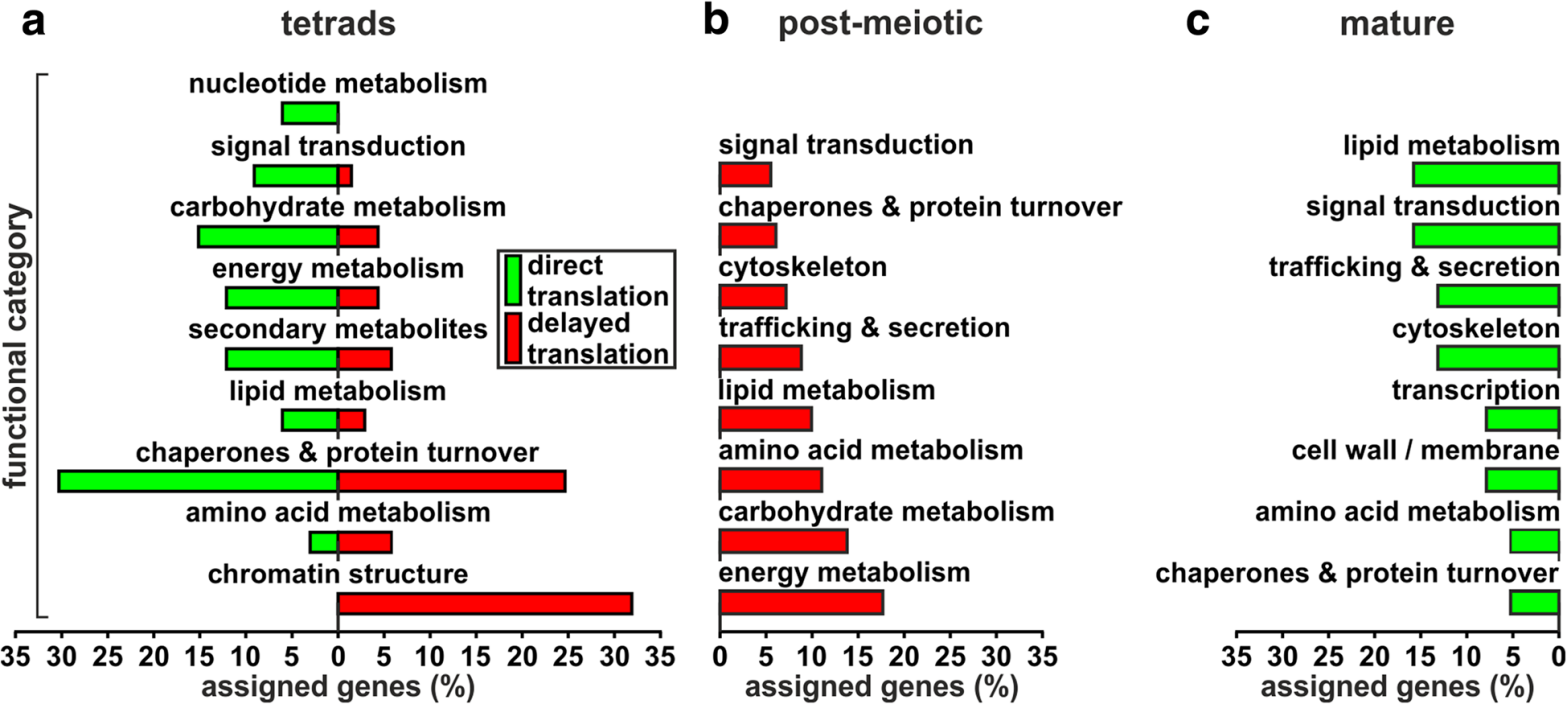
Functional annotation of genes showing direct and delayed translation. Distribution of genes with direct (green) and delayed translation (red) among functional categories for tetrads (**a**), post-meiotic (**b**) and mature pollen (**c**). Genes are considered as directly translated if transcript and protein are increased in the same stage, whereas delayed translation implies that first the transcript is increased and the protein increase is shifted by one stage. All genes assigned to at least one functional category are considered. Genes assigned to several categories are counted in each category. Categories comprising at least 5% of the genes with an assigned function are shown

In the next step, we focused on the question whether the different genes with direct and delayed translation are related to specific cellular functions. For this reason, we assigned functional categories of the eukaryotic orthologous groups (KOG) annotation (Additional file 4: Table S3) to each gene found to be directly or delayed translated during pollen development. Subsequently, we determined the distribution of the genes among the different functional categories (Fig. 3; Additional file 5: Table S4) to identify whether the different genes with direct and delayed translation are related to specific cellular functions.

The majority of the genes with direct translation in tetrads (Fig. 3a**,** green) was assigned to the category of ‘chaperones & protein turnover’ (Additional file 5: Table S4), comprising among others two Hsp90s (Hsp90–4 and Hsp90–3), one Hsp70 (BIP4) and one sHsp (Hsp23.8-MI). Interestingly, a large number of genes showing delayed translation in tetrads was classified as ‘chaperones & protein turnover’. These genes comprise one Hsp100 (ClpB3) and one Hsp70 (Hsp70–5) as well as three protein disulfide isomerases and five subtilisin-like proteases. In addition, many genes with direct translation in tetrads appear to be involved in metabolic processes as they were assigned to functional classes like ‘carbohydrate metabolism’, ‘energy metabolism’, ‘lipid metabolism’ and ‘nucleotide metabolism’ as well as ‘secondary metabolites’. In turn, genes with delayed translation in tetrads (Fig. 3a, red) are strongly enriched for ‘chromatin structure’ indicating an important role of chromatin remodeling in post-meiotic pollen. Out of the 22 genes assigned to this functional category 21 are encoding for histones. Four of them belong to the H2B family, eight to the H3 family and nine to the H4 family.

Genes with delayed translation in post-meiotic pollen (Fig. 3b) are largely involved in metabolic processes (Additional file 5: Table S4). One of these processes is ‘energy metabolism’, comprising for example genes encoding for four ATP synthase subunits and four V-type proton ATPase subunits. Another category with high assignment of genes is ‘carbohydrate metabolism’. Among these genes we observed 11 genes encoding for seven out of ten glycolysis reaction enzymes comprising a hexokinase, phosphofructokinase, fructose-bisphosphate aldolase, triosephosphate isomerase, glyceraldehyde-3-phosphate dehydrogenase, enolase and pyruvate kinase. This observation suggests a targeted translation of glycolysis proteins during the transition from post-meiotic to mature pollen.

Regarding genes showing direct translation in mature pollen (Fig. 3c), we observed the highest number of assigned genes for ‘lipid metabolism’. The next three functional categories with a high assignment are ‘signal transduction’, ‘trafficking & secretion’ and ‘cytoskeleton’. Here, we identified proteins required for interaction with the environment as well as growth and differentiation, including two Ras-related proteins, calmodulin, two kinases and a phosphatase as well as actin 4, actin depolymerizing factor 10 and myosin XI. Summarizing, we show that the direct or delayed synthesis of proteins is related to specific biological processes in a stage dependent manner.

### The transcriptome and proteome response to HS in pollen

**Fig. 4:**
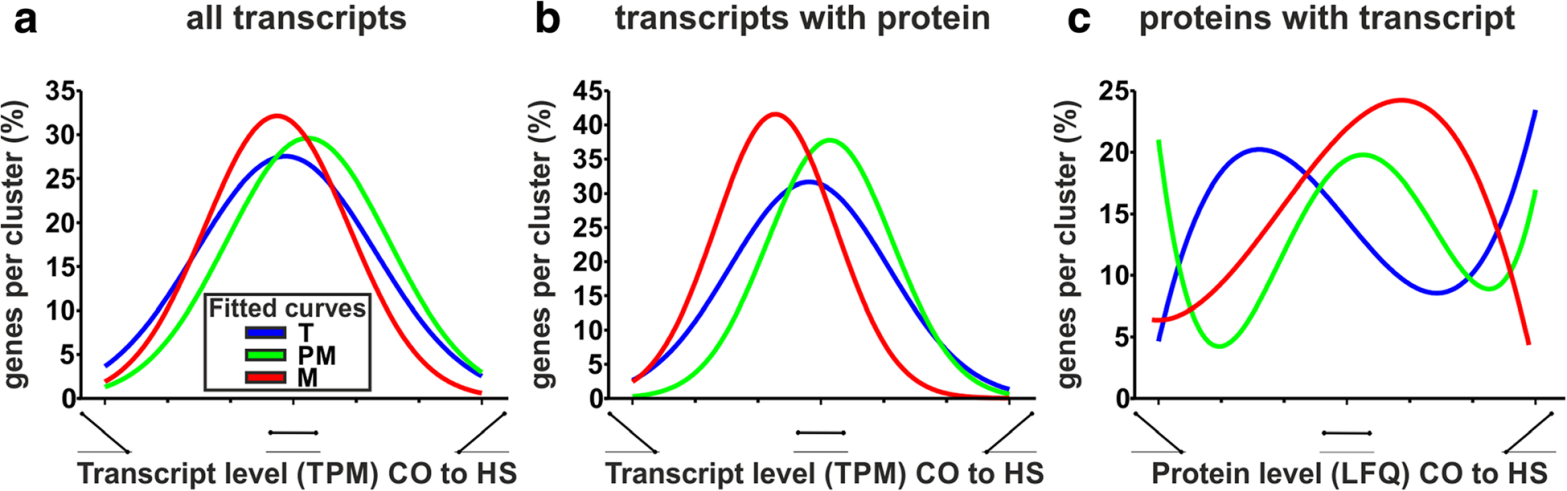
Stage dependent changes of transcript and protein abundance in response to HS. The curves represent the least square fit of percentage of genes assigned to transcript (**a** and **b**) and protein clusters (**c**) for tetrads (blue), post-meiotic (green) and mature pollen (red). Either all assigned genes (**a**) or only genes detected in transcriptome and proteome (**b** and **c**) were considered. Clusters are ordered from downregulation to upregulation after HS

We analyzed the effect of HS on the different pollen stages to examine differences in transcriptome and proteome HS response in dependency of the developmental stage. We performed k-means clustering (k = 7) on the relative transcript and protein levels across CO and HS for pollen from all three developmental stages independently (Additional file 6: Table S5). Again, proteins assigned to the same protein group were ungrouped and treated independently. To compare transcriptome and proteome alterations in response to HS, we generated an overlap matrix for each developmental stage (Additional file 1: Figure S5). By fitting the number of genes assigned to the transcript and protein clusters we observed differences in abundance changes of transcripts and proteins for all developmental stages. (Fig. 4). We defined genes from the leftmost clusters as downregulated and those from the rightmost cluster as upregulated. Genes from the remaining clusters were defined as having stable levels in response to HS across all stages.

Considering all genes with a detected transcript (Fig. 4a) we observed a Gaussian distribution centered on the cluster with steady state levels for all developmental stages. As previously described about 5% of the plant transcriptome is enhanced upon HS [37]. Similarly, ~ 3% of the genes in tetrads and post-meiotic pollen are assigned to the cluster showing strong upregulation upon HS, whereas less than 1% of the genes in mature pollen were assigned to this cluster. Regarding genes downregulated upon HS, we observed for tetrads around 4% assigned genes and for post-meiotic and mature pollen ~ 2% assigned genes. Thus, only up to 7% of all detected genes showed a strong effect upon HS in the single developmental stages of pollen.

Next, we focused on those genes detectable at transcriptome and proteome level of the pollen stages to have a common starting point for the comparison of the transcriptome and proteome response (Additional file 6: Table S5 highlighted in bold). For tetrads and post-meiotic pollen we observed that approximately 1% of the genes showed an increase of transcript abundance in response to HS (Fig. 4b), whereas this was not apparent for mature pollen. Interestingly, there was no strong downregulation in post-meiotic pollen, but a downregulation for around 2.5% of the genes in tetrads and mature pollen. Surprisingly, we did not observe a Gaussian distribution for any developmental stage at the proteome level but a high number of genes, with protein levels strongly affected by HS (Fig. 4c). Approximately 20% of proteins showed accumulation in response to HS in tetrads and post-meiotic pollen, while only 4% showed similar behavior in mature pollen. Furthermore, the number of proteins showing a reduced abundance in response to HS was higher in post-meiotic pollen (21%) compared to tetrads and mature pollen (~ 5% each).

**Fig. 5:**
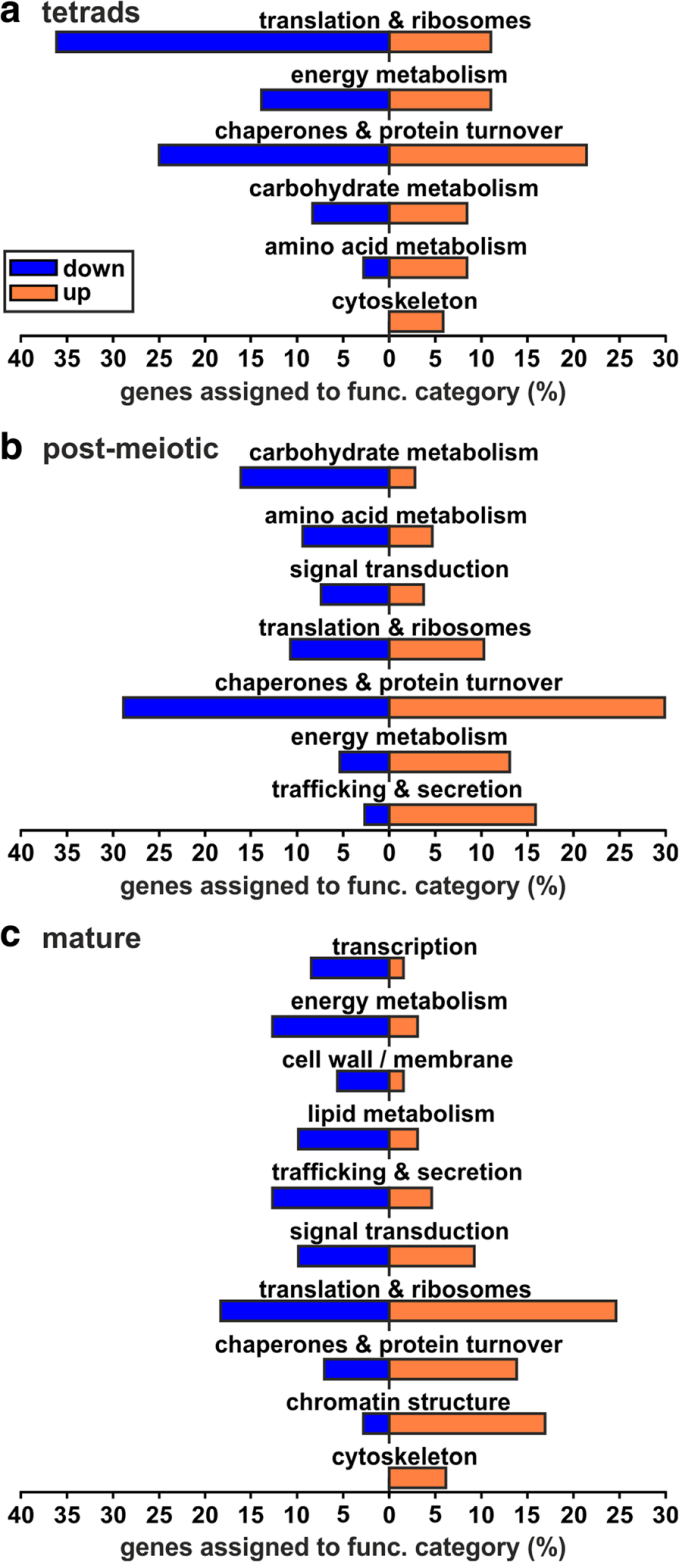
Functional categories affected by HS. Distribution of proteins down- (blue) and upregulated (orange) after HS among functional categories for tetrads (**a**), post-meiotic (**b**) and mature (**c**) pollen. Proteins assigned to at least one category were considered and could be counted more than once. Categories comprising at least 5% of the proteins with an assigned function are shown

Next, we analyzed the genes with a protein assigned to clusters with strong down- and upregulation (left- and rightmost clusters in Fig. 4c, Pr1 and Pr7 clusters in Additional file 1: Figure S5). Regarding strong downregulation we observed the minimum number of genes in tetrads (47 genes) and maximum in post-meiotic pollen (159 genes), while the number of strongly upregulated genes was lowest in mature (69 genes) and highest in tetrads (159 genes). To analyze the down- and upregulated proteins in more detail, we classified the proteins based on the KOG annotation (Fig. 5; Additional file 7: Table S6). Many proteins that show enhanced or reduced levels in response to HS belong to the ‘chaperones & protein turnover’ class (Additional file 8: Table S7). This holds true for all developmental stages while particularly the two earlier stages are enriched in Hsps. In both stages seven proteins of the sHsp family accumulated under HS, while three Hsp60, one Hsp70, one Hsp90 and one Hsp100 accumulated in tetrads but had reduced levels in post-meiotic pollen. In addition, two Hsp100s and two Hsp40s accumulated solely in tetrads and three sHsps and one Hsp40 solely in post-meiotic pollen. Further, four Hsp60 and two Hsp90s were less abundant in post-meiotic pollen after HS when compared to CO. In mature pollen by and large no effect of HS on the abundance of the Hsp family members was observed. In addition to chaperones, seven proteasome subunits solely accumulated in post-meiotic pollen, one in post-meiotic and mature pollen and another one solely in mature pollen. In turn, we observed one proteasome subunit with lower level after HS in tetrads, one in post-meiotic pollen and one in tetrads and mature pollen. For three proteasome subunits we observed an opposite HS effect among the developmental stages. The first subunit accumulated in post-meiotic pollen but was reduced in mature pollen, the second was reduced in post-meiotic pollen but accumulated in mature pollen and the third was reduced in tetrads but accumulated in mature pollen.

A second enriched functional category is the ‘translation & ribosomes’ class (Fig. 5; Additional file 8: Table S7). Most of the enhanced or reduced proteins in response to HS either encode for eukaryotic translation initiation factors (eIFs) or eukaryotic ribosomal proteins (RPs). These proteins are known to be altered upon HS leading to changes in translation initiation and composition of ribosomes [38–40]. Five eIFs accumulated in tetrads, of which one was reduced in post-meiotic pollen and two also accumulated in mature pollen. Further, three eIFs are enhanced in post-meiotic pollen as well as one reduced in post-meiotic and three in mature pollen.

Even a stronger difference between the developmental stages was apparent for the eukaryotic RPs. The accumulation of these proteins occurs in a developmental stage-specific manner. Four RPs have higher protein levels after HS in tetrads (L5, L28, S8 and S19), six in post-meiotic (L6, L14, S4, S8, S15 and S18) and eight in mature pollen (2xL7A, 3xL24 and 2xS25). In addition two RPs (2xL19) have enhanced levels in tetrads and mature pollen. Abundance reduction of RPs was as well stage-specific, except for one RP (L8) affected in all three developmental stages and two in tetrads and mature pollen (L18 and S8). In addition, eight are lower abundant after HS in tetrads (L6, 2xL12, L13, 3xS5 and S7), six in post-meiotic (3xL10, 2xL21 and S12) and five in mature pollen (4xS13 and S15).

Further, we observed some categories preferentially regulated in a specific developmental stage (Additional file 7: Table S6). For example in post-meiotic pollen we observed a strong reduction of protein levels of factors of the ‘carbohydrate metabolism’ class (Fig. 5b blue) including six genes encoding for four enzymes of glycolysis (glucose-6-phosphate isomerase, phosphoglycerate mutase, enolase and pyruvate kinase). In addition, a downregulation of polysaccharide degrading enzymes like a beta-glucosidase, a beta-galactosidase and two beta-fructofuranosidases could be detected. This observation suggests a downregulation of sugar catabolism in post-meiotic pollen after exposure to HS. In addition, we observed an increase of the protein levels of genes belonging to ‘trafficking & secretion’ in post-meiotic pollen (Fig. 5b orange). These genes include four small G proteins as well as a Ran GTPase binding protein. In mature pollen, particularly proteins related to ‘chromatin structure’ family accumulate in response to HS (Fig. 5c orange). Two are histones of the H2B family and eight histones of the H3 family suggesting a remodeling of chromatin upon HS in mature pollen.

### HS-induced regulation of Hsps and Hsfs at transcriptome and proteome level in pollen

**Fig. 6:**
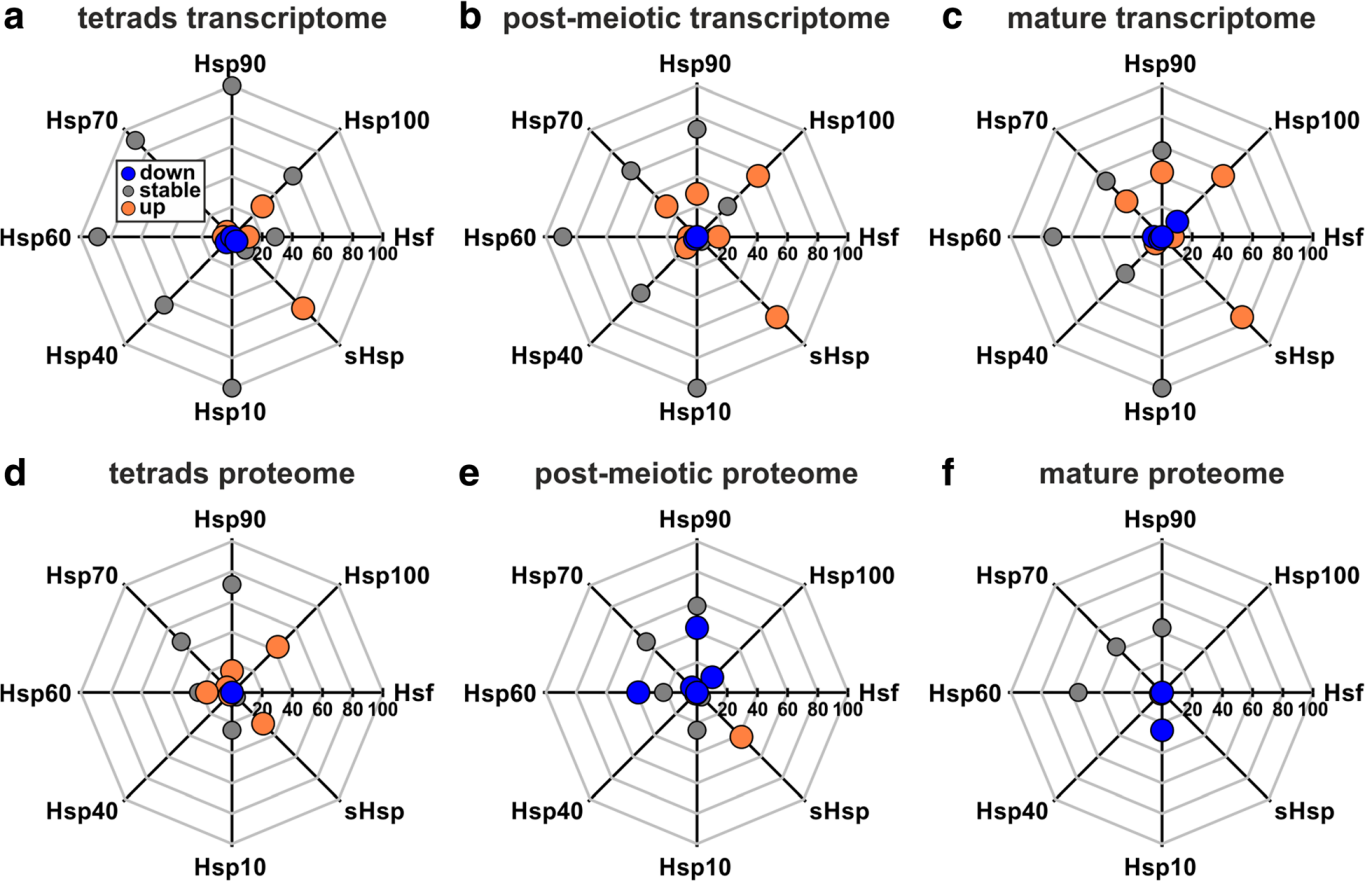
Regulation of Hsp and Hsf families upon HS at transcript and protein level. Shown is the percentage of Hsp and Hsf family proteins detected in tetrads (**a** and **d**), post-meiotic (**b** and **e**) and mature pollen (**c** and **f**) at transcript (upper row) and protein level (lower row). Proteins of the different families are subdivided with respect to their regulation upon HS with either downregulation (blue), stable levels (grey) or upregulation (orange)

Among the genes down- and upregulated at the proteome level under HS we identified several Hsps. As these genes comprise well-known key players of HS response, we analyzed the response of the different Hsp and Hsf families to HS at both transcriptome and proteome level in more detail. We categorized all Hsps and Hsfs detected by transcriptome clustering (Additional file 6: Table S5) as downregulated (cluster Tr1), not altered (stable, clusters Tr2 to Tr6) or upregulated (cluster Tr7) for all developmental stages. We additionally classified them based on their proteome clustering (Additional file 6: Table S5) as reduced (down, cluster Pr1), not altered (stable, clusters Pr2 to Pr6) or accumulated (up, cluster Pr7). After categorization we determined for each family the fraction of detected members within the classified sets for all developmental stages (Fig. 6; Additional file 9: Table S8).

For tetrads we observed that at the transcriptome level all seven annotated Hsp90s and all four annotated Hsp10s are not altered after HS (Fig. 6a). Similarly, more than 60% of the annotated Hsp40s, Hsp60s and Hsp70s showed stable transcript levels and only small fractions were down- or upregulated. For the Hsp100 and sHsp family a higher fraction is regulated in response to HS with 29 and 67% upregulated members, respectively. Upregulated Hsp100s included ClpB1 and ClpB4. For the Hsf family we detected only ~ 40% of all members, whereby 10% were upregulated and 30% showed stable transcript levels. The upregulated Hsfs included HsfA2, HsfA7 and HsfB2b.

In the proteome of tetrads we observed a smaller fraction of detected Hsps and Hsfs (Fig. 6d) when compared to the transcriptome (Fig. 6a). In comparison to the transcriptome we observed only a single Hsp10 (Cpn21-P) in the proteome with stable levels. Further, we identified six of the seven Hsp90s in the proteome, whereby one of them accumulated at the proteome level but was stable at the transcriptome level (Hsp90–1). Interestingly, we identified only ~ 40% of the Hsp60s and Hsp70s detected in the transcriptome in the proteome, from which CCT-δ, Cpn60 (3), Cpn60-β (2) and Hsp70–13 had elevated protein levels under HS. A higher fraction of upregulated proteins was observed for the sHsp and Hsp100 family. Seven sHsps accumulated at the proteome level and were also upregulated at the transcriptome level. In the case of the Hsp100 family we detected an accumulation of ClpB1, ClpB3 and ClpC2, whereby the latter two were not upregulated at the transcriptome level.

We identified for all Hsp families at least 60% of the annotated representatives in the transcriptome of post-meiotic pollen (Fig. 6b). Further, we observed a substantial upregulation for members of the Hsp70, 90 and 100 as well as sHsp family ranging from 28% (Hsp70 and Hsp90) to 75% (sHsp) of all annotated members. These upregulated Hsps included Hsp70–5, Hsp70–6, Hsp70–8, Hsp70–10, BIP2 and BIP3 of the Hsp70 family, Hsp90–3 and Hsp90–4 of the Hsp90 family and ClpB1, ClpB3, ClpB4 and ClpD of the Hsp100 family as well as 18 sHsps. Regarding the Hsf family, we observed an upregulation of HsfA2, HsfA3, HsfB1 as well as HsfB2b. While we detected predominantly an upregulation of different Hsp families at the transcriptome level, we observed downregulation at the proteome level, especially for the Hsp60 and Hsp90 family (Fig. 6e). Interestingly, all Hsp60s and Hsp90s with downregulated protein levels had stable transcript levels, except for Hsp90–3 for which an enhanced transcript level was observed. Only for 42% of the sHsps we found higher protein levels in response to HS. These 10 sHsps were upregulated at the transcriptome level as well.

In mature pollen we observed a substantial amount of members of the Hsp70, Hsp90, Hsp100 and sHsp family with upregulated transcript levels (Fig. 6c). The fraction of upregulated members ranged from 33% (Hsp70) up to 75% (sHsp) and included Hsp70–5, Hsp70–6, Hsp70–8, Hsp70–10, Hsp70/110–1, Hsp70/110–2 and BIP3 of the Hsp70 family, Hsp90–3, Hsp90–4 and Hsp90–5 of the Hsp90 family, ClpB1, ClpB3, ClpB4 and ClpD of the Hsp100 family and 18 sHsps. For the Hsf family we detected four members, whereby HsfA2 and HsfB1 were upregulated and HsfA4c and HsfA5 not affected by HS. The remaining Hsp families were not represented by changes of transcript levels under HS. At the proteome level we detected only a small number of Hsps (Fig. 6f). For the Hsp60, Hsp70 and Hsp90 family we detected between 43% (Hsp70 and Hsp90) and 56% (Hsp60) of the annotated members, whereby the protein levels were not affected by HS. Only downregulation of Cpn21-P of the Hsp10 family and upregulation of a DnaJ-C of the Hsp40 family was observed.

### General and stage-specific HS regulation of Hsps and Hsfs in pollen

We observed common as well as stage-specific HS induced regulations of Hsps and Hsfs at the transcriptome level (Fig. 6a to c). For instance, we observed an upregulation of HsfA2 in all developmental stages, of HsfB2b in tetrads and post-meiotic pollen, of HsfB1 in post-meiotic and mature pollen, of HsfA7 only in tetrads and of HsfA3 only in post-meiotic pollen. We also observed a stage-specific behavior for HsfA1a, the described master regulator of thermotolerance in tomato [41], which had stable transcript levels in tetrads and post-meiotic pollen after HS but was not detected under CO or HS in mature pollen. In turn, sHsps appear to be globally upregulated with 15 members upregulated in all stages, three in post-meiotic and mature pollen as well as one exclusively in tetrads. Alike, ClpB1 and ClpB4 of the Hsp100 family are upregulated in all stages, while ClpB3 and ClpD are solely upregulated in post-meiotic and mature pollen, respectively. Strong differences between the stages were observed for the Hsp70 family. Only a single Hsp70 was upregulated in all stages, whereas four were upregulated in post-meiotic and mature pollen and three either in post-meiotic or mature pollen. Similarly, no Hsp90 is upregulated in tetrads, two (namely Hsp90–3 and Hsp90–4) in post-meiotic and mature pollen and one (Hsp90–5) in mature pollen only.

At the proteome level we observed strong differences between the developmental stages. For example, we detected no sHsps in mature pollen but seven sHsps upregulated in tetrads and post-meiotic pollen and three exclusively upregulated in post-meiotic pollen. Surprisingly, the three Hsp60s upregulated in tetrads were downregulated in post-meiotic pollen together with additional four Hsp60s. Downregulation of Hsp60s was not observed in tetrads and mature pollen and seems to be specific for post-meiotic pollen. Additional post-meiotic specific downregulation was observed for members of the Hsp90 and 100 family. The downregulation included Hsp90–3, which had stable protein levels in tetrads and was not detected in mature pollen, as well as Hsp90–1 and ClpB3 that interestingly were upregulated in tetrads. In addition, solely in tetrads we observed an upregulation of ClpB1 and ClpC2. The comparison of the stages has revealed at least for the transcriptomes for some of the families shared regulation among the stages (e.g. sHsp) but also stage dependent regulation of families (e.g. Hsp70). In contrast, at the proteome level nearly no shared regulation between the stages was observed.

## Discussion

In this study, we combined MACE and LC-MS/MS experiments to elucidate the transcriptome and proteome of developing tomato pollen under normal growth conditions and after exposure to elevated temperature. Our analyses revealed drastic changes in transcriptome and proteome complexity throughout pollen development and further demonstrated the existence of two translational modes active in developing pollen. The translational modes were defined as direct and delayed translation and shown to possess a preference for specific functional processes within the individual developmental stages. In addition, we provide evidence for a varying HS response between the stages, which became apparent in the number and composition of differentially regulated proteins. Here, especially proteins related to protein synthesis (eIFs and RPs), maintenance (Hsps) and degradation (proteasome subunits) showed an alternative regulation among the developmental stages.

### Transcriptome and proteome alterations during pollen development

We could show that the transcriptome of developing tomato pollen comprises about 12,600 transcripts across the three analyzed stages (Additional file 1: Figure S2A). This number is comparable to the 14,000 genes expressed along the development of pollen in *A. thaliana* [3]. Similar to *A. thaliana* and *O. sativa* [3, 5], we observed a decrease of the transcript number from early (~ 11,000 transcripts in tetrads) to late developmental stages of tomato pollen (~ 5000 transcripts in mature pollen; Table 1). In addition, the transcriptome is largely stage specific as observed by PCA analysis (Fig. 1a). The proteome size we identified for tomato pollen was 1000 protein groups along the course of development (Additional file 1: Figure S2A). This number is smaller than the previously reported 1821 proteins [10]. Possible reasons for this difference could be (i) the difference in the selection of developmental stages (polarized microspores were not analyzed in here), (ii) our high stringency in protein detection (detected in at least two out of three replicates) or (iii) the grouping of proteins into protein groups. With regard to the proteome size of the individual stages we observed an increase of the proteome size from tetrads to mature pollen (Table 1), which is consistent with earlier observations [10].

### The functional relevance of direct and stage-delayed translation in developing pollen

**Fig. 7:**
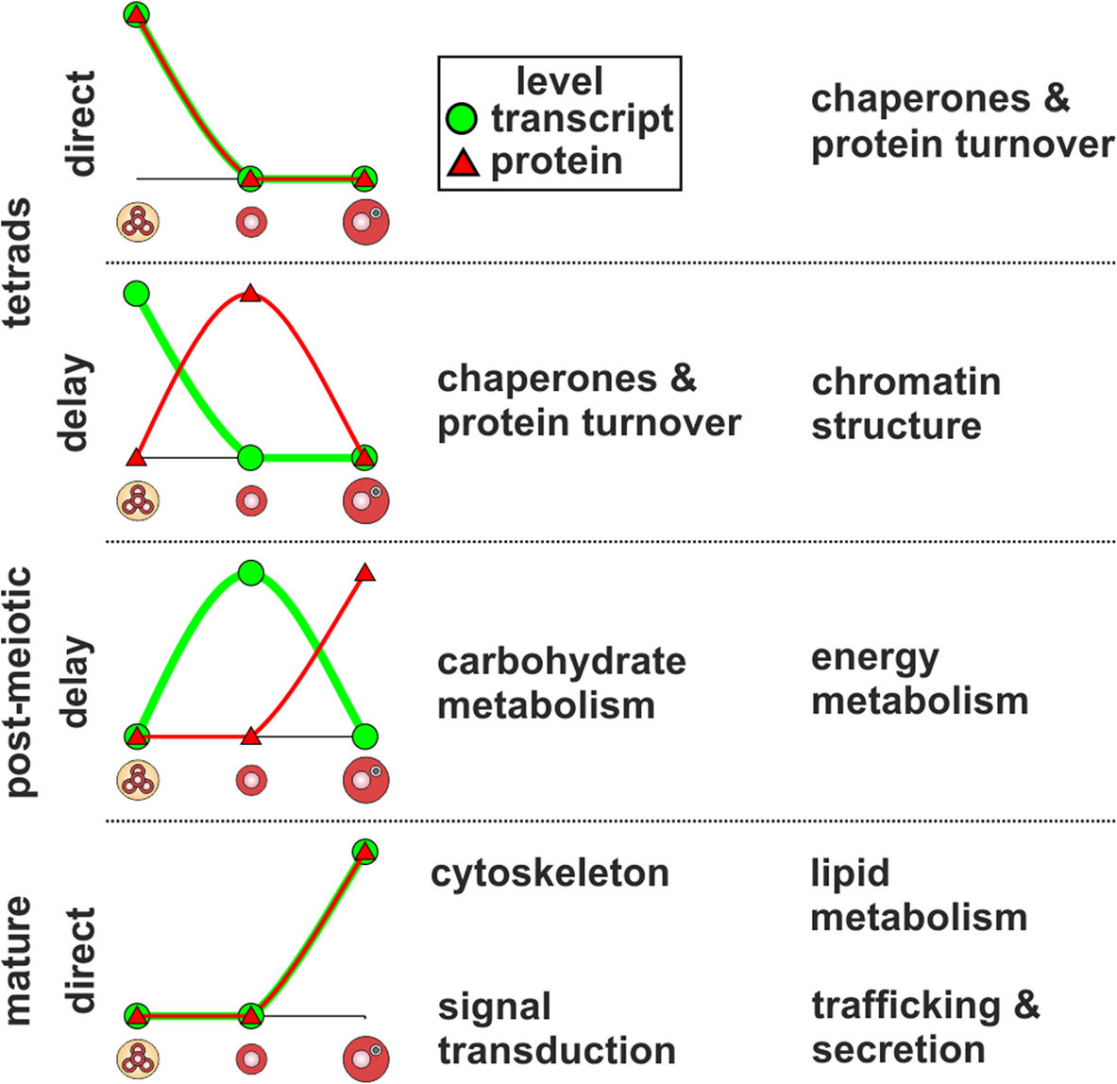
Functional categories and proteins important for development of pollen. Shown are functional categories enriched for genes showing direct or delayed translation in the different pollen stages together with the corresponding transcript and protein levels across the pollen stages

The comparison of the stage-increased transcriptomes and proteomes revealed the modes of direct and delayed translation (Fig. 2a and b). The majority of genes with direct translation in tetrads was assigned to the ‘chaperones & protein turnover’ category (Fig. 7 tetrads direct; Additional file 5: Table S4) including a sHsp, BIP4 of the Hsp70 family as well as two Hsp90s. This finding supports the idea of developmental priming, which aims to protect developing pollen against possible abiotic stresses by the expression of stress-responsive genes like Hsps [10]. Further BiP proteins of the Hsp70 family play an essential role in the correct development of early tomato pollen stages [42]. Similarly, also for genes with delayed translation we observed high assignment to ‘chaperones & protein turnover’ (Fig. 7 tetrads delay; Additional file 5: Table S4). Among these genes we identified a member of the Hsp100 and a member Hsp70 family, which might contribute to a developmental priming of post-meiotic pollen. However, the majority of genes with delayed translation in tetrads was assigned to ‘chromatin structure’ including multiple members of the histone families H2B, H3 and H4. Next to histone modifications, the expression and incorporation of different histones into nucleosomes regulates DNA accessibility and by this affects gene expression with a strong effect on developmental processes [43]. Further supporting a potential role of histones in the development of pollen is the developmental and organ specific expression of different H3 variants in *Zea mays* and *O. sativa* [44].

Proteins accumulating in mature pollen by either direct or delayed translation were assigned to different metabolic processes as well as ‘cytoskeleton’, ‘signal transduction’ and ‘trafficking & secretion’ (Fig. 7 post-meiotic delay and mature direct; Additional file 5: Table S4). The enrichment of proteins related to ‘energy metabolism’ and ‘carbohydrate metabolism’ (Fig. 7**,** post-meiotic delay) was also described for mature pollen of *O. sativa* [11], whereas an enrichment of these functions was never detected at the transcriptome level [2, 10]. This phenomenon can be explained by our observation that the transcripts encoding these proteins are synthesized in post-meiotic pollen but translated with a delay during the transition from post-meiotic to mature pollen. Proteins embedded in these functional categories included seven out of ten glycolytic enzymes as well as several ATP synthase subunits reflecting a preparation for the high energy demand of germination and pollen tube growth of mature pollen [45].

The absence of directly translated genes in the post-meiotic pollen and delayed translated genes in the mature pollen results from our definition and experimental set up. In the first case, direct translation was not observed as the number of genes from clusters with post-meiotic increased transcript or protein levels (PM increased in Fig. 2a and b) that fulfilled our criterion of direct translation was too low to exceed our threshold (scaled value of at least 0.5) and by this they were not further analyzed. In the latter case, we did not have proteome information from pollen during or after pollen tube formation, which would be the subsequent stage.

### Pollen viability at elevated temperature

Regarding the response of the individual stages to elevated temperature, we found that mature pollen was the least responsive stage. This finding supports the general assumption that stages after pollen mitosis are relatively tolerant to HS [17]. The different strength of the HS response was most apparent at the proteome level. The number of proteins changed in their abundance is drastically higher for tetrads (~ 30%) and post-meiotic pollen (40%) than for mature pollen (9%, Fig. 4, Additional file 1: Figure S5). In contrast, we observed for all three stages only minor changes at the transcriptome level (Fig. 4), whereby the percentage of upregulated transcripts is comparable to the observed 5% for plant transcriptomes [37].

The different strength in the regulation of transcriptome and proteome in response to elevated temperature supports the general assumption of a low correlation between transcript and protein levels, which was recently also shown for the HS response of soybean roots [46]. Reasons for a low correlation between transcript and protein levels are diverse. On the one hand, the half-life of proteins and their corresponding transcripts can differ [47], which for instance can lead to an earlier decrease of transcript levels towards their initial state. On the other hand, the regulation of translation initiation either enhancing or reducing protein synthesis decouples transcript and protein levels [48]. Thus, the description of response networks requires both, the proteome and the transcriptome level. The proteome level gives an information about the existing response reaction and the transcript level can provide insights into acquired mechanisms.

### The effect of elevated temperature on genes with direct and delayed translation

**Fig. 8:**
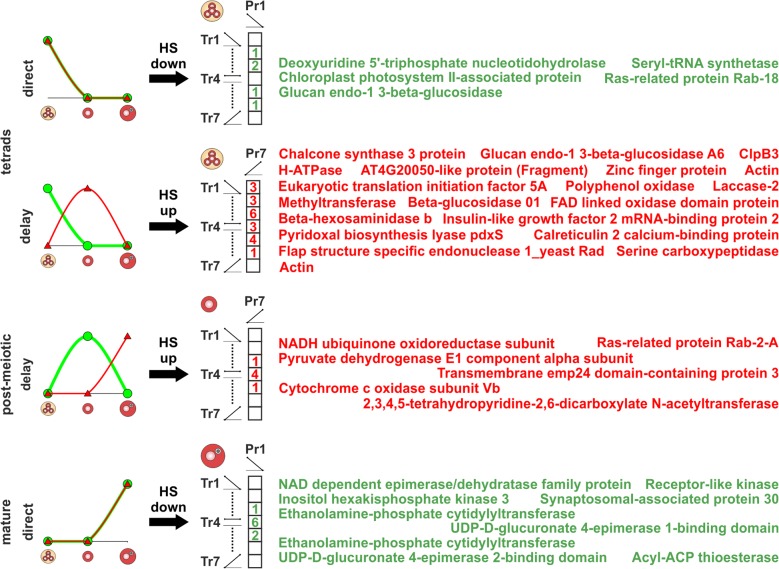
Effect of HS on transcript and protein levels of genes with direct or delayed translation. Genes with direct (green) and delayed translation (red) in tetrads (row 1 and 2), post-meiotic (row 3) and mature pollen (row 4) were analyzed for their regulation at transcriptome and proteome level in response to HS. For this purpose, the genes were traced in the transcript (Tr) and protein clusters (Pr) derived from the clustering of transcript and protein levels across CO and HS. Genes with direct translation were analyzed for HS induced reduction of protein levels (Pr1) and genes with delayed translation for HS induced increase of protein levels (Pr7). Numerals in the cells indicate the number of genes detected in a combination of transcript (Tr1 to Tr7) and protein cluster (Pr1 or Pr7). In addition, the functional description of the genes with HS induced reduction or accumulation of protein levels is shown

When analyzing the effect of HS on the protein level of genes with direct and delayed translation we observed for most of the genes no changes in protein abundance after HS. However, for five genes with direct translation in tetrads (Fig. 8 tetrads direct; 54 in total) and nine with direct translation in mature pollen (Fig. 8 mature direct; 53 in total) we observed a drastic reduction of protein levels (Pr1) but no effect on transcript levels (Tr2 to Tr6). Among the five genes with direct translation and HS induced reduction of protein levels in tetrads we identified a glucan endo-1 3-beta-glucosidase (Fig. 8 tetrads direct). This protein is related to cell wall metabolism and was shown to accumulate in response to drought in roots or leaves of a tolerant genotype of wheat, whereas in a sensitive genotype the reduction of the protein was observed [49]. These findings match our observation for tetrads, pointing towards an increased sensitivity of tetrads to abiotic stresses and further emphasizes the assumption of a higher sensitivity of early developmental stages [17]. Among the nine genes with direct translation and HS induced reduction of protein levels in mature pollen we observed two UDP-D-glucuronate 4-epimerases (Fig. 8 mature direct). These proteins belong to one of 11 NDP-sugar-interconversion enzymes families [50] and are required for the synthesis of UDP-D-galacturonate, which is an activated precursor for the synthesis of pectins [51]. Downregulation of these proteins after HS might impair pectin biosynthesis in mature pollen, which is in turn critical for cell wall integrity as well as pollen tube growth [52] and by this could contribute to the reduced germination rate of heat-stressed pollen [53]. Comparable results were shown for members of the UDP-Glucose 4-Epimerase family (UGE), where uge2 and uge3 pollen was incapable of pollination [54].

Regarding the effect of HS on the protein level of genes with delayed translation, we observed for 20 genes with delayed translation in tetrads (Fig. 8 tetrads delay; 108 in total) and six with delayed translation in post-meiotic pollen (Fig. 8 post-meiotic delay; 223 in total) increased protein levels (Pr7) in response to HS but no increase in transcript abundance (Tr1 to Tr6). Among the 20 genes with delayed translation and HS induced increase of protein levels in tetrads we observed ClpB3 (Fig. 8 tetrads delay). ClpB3 is a plastid localized member of the Hsp100 family and plays an important role in the HS response of plastids [55]. Induction of ClpB3 transcript levels upon HS is a general mechanism and was for example described for different organs of *A. thaliana* seedlings [56] and mixed developmental stages of tomato pollen [57]. Surprisingly, for tetrads we observed only a HS induced increase of ClpB3 protein but not of transcript levels, whereas for post-meiotic and mature pollen we observed the transcript increase (Additional file 9: Table S8). We conclude that ClpB3 transcripts present in tetrads but preserved for a delayed translation during the transition from tetrads to post-meiotic pollen, switch to a direct translation during HS. Similarly, we observed for a cytochrome c oxidase and a NADH ubiquinone oxidoreductase subunit, which belong to the genes with delayed translation in post-meiotic pollen, a HS induced upregulation of protein levels in post-meiotic pollen (Fig. 8 post-meiotic delay). Both cytochrome c oxidoreductase and NADH ubiquinone oxidoreductase subunits were shown to have elevated transcript levels in response to HS due to an enhanced respiratory activity [58]. This finding suggests that instead of synthesizing new transcripts upon HS, post-meiotic pollen utilize pre-synthesized transcripts of genes with delayed translation for a direct translation in response to HS.

### The role of protein synthesis, maintenance and degradation in the HS response of pollen

**Fig. 9:**
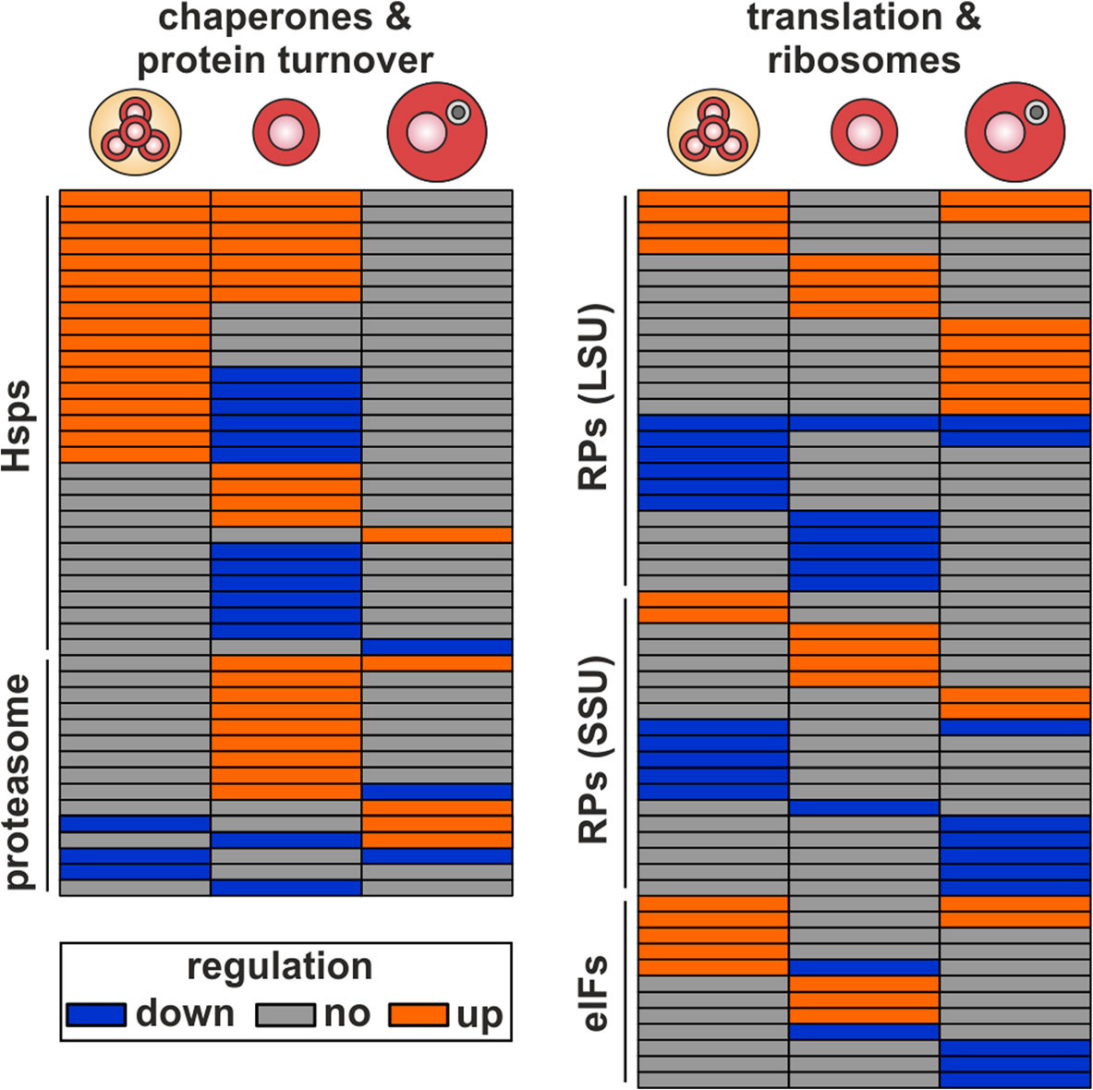
HS response of the chaperone network, the proteasome and the ribosome in pollen. Shown is the HS regulation of proteins belonging to ‘chaperones & protein turnover’ and ‘translation & ribosomes’ is shown for the three developmental stages. Proteins include different Hsps (Hsps), proteasome subunits (proteasome), RPs of the large (LSU) and small subunit (SSU) and eIFs

Most affected during heat stress were proteins assigned to ‘chaperones & protein turnover’ or ‘translation & ribosomes’ (Fig. 9; Additional file 8: Table S7). Within these categories we observed several Hsps, proteasome subunits, RPs as well as eIFs, which hints towards a central role of protein synthesis, maintenance and degradation in the response of pollen to HS.

The upregulation of the 26S proteasome complex in response to stress is described for plants in general [59]. However, a HS induced upregulation of proteasome subunits occurs only in post-meiotic pollen, but not in tetrads or mature pollen.

The abundance of RPs [40] and eIFs [60] generally changes in response to several stresses, including phosphate- and iron-deficiency as well as cold, heat and oxidative stress. Further, eiFs [61] as well as altered ribosome composition [40] were shown to influence selective mRNA translation under stress conditions. The presence of stress-responsive elements, including heat shock elements, was shown in the promoter regions of RPs and might function as starting points for the differential regulation of RPs by Hsfs [39]. The down- and upregulation of RPs as well as eIFs is largely stage-specific (Fig. 9), which might lead to alterations in the translation machinery in the developmental stages and by this contributes to a stage-specific HS responses. For instance in post-meiotic pollen, we observed a downregulation of RPL10 proteins after HS (Additional file 8: Table S7). Interestingly, mutation of *AtRPL10C* (one of the three RPL10 genes in *A. thaliana*) leads to abundance changes of different proteins under UV-B stress, whereby the majority of the proteins is reduced [62]. This finding suggests that the observed reduction of RPL10 proteins in post-meiotic pollen might lead to reduced translation of prioritized mRNAs, which could contribute to the high degree of downregulated proteins in post-meiotic pollen after HS (Fig. 4c). A similar model was proposed by Sormani et al. [63], where the authors propose that the downregulation of a subset of cytosolic RPs under stress leads to reduced translation of certain mRNAs.

After HS several Hsps are accumulated in tetrads and post-meiotic pollen. Additionally, in post-meiotic pollen several Hsps show lower abundance than under CO. In mature pollen, nearly no regulation of Hsps could be observed (Fig. 9; Additional file 8: Table S7). The comparison of the alterations of Hsps at transcript and protein levels in response to HS revealed strong differences especially for post-meiotic and mature pollen (Fig. 6; Additional file 9: Table S8). For instance, in post-meiotic pollen we observed a high degree of downregulated Hsps except for members of the sHsp family, although the transcript levels of these Hsps were not affected or even upregulated under HS. In mature pollen Hsps were found to be enhanced at transcriptional but not at protein level (Fig. 6; Additional file 9: Table S8). We conclude that at the transcriptome level the developmental stages react more or less similar, whereas significant alterations exist at the proteome level. Here, tetrads showed the most active HS response, whereas for post-meiotic and mature pollen only a diminished or no response, respectively, was observed. These latter findings are in accordance with the general assumption of a diminished Hsp activation in mature pollen after HS [17].

It is well established that Hsfs regulate the heat stress response in plants [23]. However, as transcription factors are low abundant proteins they largely escaped proteomic detection [24]. The ‘master regulator’ HsfA1a [24, 64, 65] was detected at least at the transcriptome level in tetrads and post-meiotic pollen (Additional file 9: Table S8). After application of elevated temperatures HsfA2 is present in all three analyzed stages. Interestingly, other Hsfs seem to respond to HS in a stage-specific manner. While HsfB1 appears in post-meiotic and mature pollen, HsfB2b occurs in tetrads and post-meiotic pollen. In addition, HsfA7 (tetrads) and HsfA3 (post-meiotic) are even more stage-specific.

## Conclusions

The development of pollen is accompanied by drastic changes in the composition and sizes of transcriptome and proteome. Here, we identified two translational modes active in developing pollen, namely direct and delayed translation. Delayed translation might in part explain the weak correlation of transcriptome and proteome of different pollen stages [11, 12], as transcripts are synthesized but not directly translated into proteins in the same stage. Similarly, delayed translation could explain the under-representation of transcripts and simultaneous over-representation of proteins related to “energy metabolism” and “carbohydrate metabolism” in mature pollen [11, 12], as both processes were enriched among the genes with delayed translation in post-meiotic pollen (Fig. 7, post-meiotic delay). Further, pollen responds to elevated temperatures mainly at the proteome level, whereas the response of the transcriptome is rather marginal. Proteins differentially regulated at elevated temperatures are primarily related to synthesis, proper folding and degradation of proteins (Fig. 9). Taken together these findings suggest that, except for Hsfs and Hsps, during HS protein levels are regulated by an interplay of protein synthesis and degradation than by down- or upregulation of transcript levels.

## Methods

### Plant material, stress treatment and pollen analysis

Tomato plants (*Solanum lycopersicum* cv. Red Setter) were in the glasshouse facility of ALSIA – Research Center Metapontum Agrobios (Metaponto, Italy) grown under controlled conditions (12 h light-dark cycle with a day temperature of 25 °C and night temperature of 18–20 °C, and relative humidity at 65–70%). The solar radiation in the greenhouse was supplemented to at least 190 μmol photons m^− 2^ s^− 1^ after the appearance of the first inflorescence.

Treatments were performed as described by Bokszczanin et al. [66]. Plants were transferred in a preheated growth chamber and exposed to 38 °C for 1 h (Photosynthetically active radiation (PAR) = 85,927 ± 4,7 μmol photons m^− 2^ s^− 1^). Following the treatment, the temperature was gradually decreased to 25 °C within 30 min and plants were allowed to recover for an additional hour at 25 °C. Untreated plants were kept in the growth chamber for the same time period at 25 °C. Flower buds and open flowers were harvested 1.5 h after HS or at the same time from control plants.

As described by Bokszczanin et al. [66] tomato flowers buds were sorted according to the corresponding pollen stages as follow: 4–6 mm length for tetrad (T) (meiotic stage/microspore mother cell), 6–8 mm in length for post-meiotic (PM) stage (microspores), and > 10 mm in length for mature (M) (bicellular pollen) pollen grains.

Sepals, petals and the stigma and the style were removed using forceps. Anthers containing pollen were immersed in chilled germination solution [KNO_3_ (1 mM); Ca(NO_3_)_2_ (3 mM); MgSO_4_ (0,8 mM), H_3_BO_3_ (1,6 mM)] and kept on prior to pollen isolation and purification.

Pollen was isolated from anthers by squeezing the stamens with a 200 μl pipette tip in the germination solution, followed by vortexing for 15 s. Pollen was passed through gauze cloth, washed twice with germination solution, and centrifuged at 4 °C for 2 min at 100×g, followed by short spin to sediment the isolated pollen grains. The pellet was resuspended in 200 μl of germination solution, followed by centrifugation at 100 g for 2 min at 4 °C, and followed by short spin. The supernatant was discarded and samples preserved in liquid nitrogen. Prior to centrifugation the pollen stage and purity were confirmed using staining with 1 μg mL^− 1^ DAPI and visualized under a fluorescent microscope.

Control and heat-stressed pollen samples were collected from three independent experiments performed during three consecutive days. Samples derived from one day were treated as biological replicates.

### RNA isolation and MACE sequencing

RNA was isolated from two biological replicates per developmental stage and condition using the Macherey-Nagel NucleoSpin miRNA isolation kit according to manufacturer’s protocol. From the two resulting fractions the one comprising the large RNA (> 200 nt) was used for the next steps. MACE libraries were prepared based on the protocol of Bokszczanin et al. [66] using the proprietary TrueQuant technology for elimination of PCR bias [34]. Poly-adenylated RNA was extracted with Dynabeads mRNA Purification kit (Life Technologies) and reverse transcribed with SuperScript Double-Stranded cDNA Synthesis Kit (Life Technologies) using biotinylated poly(dT) primers. Fragmentation of cDNA was conducted with Bioruptor (Diagenode) which yielded an average size of 250 bp. Biotinylated cDNA ends were captured by Dynabeads M-270 Streptavidin Beads (Life Technologies) and ligated with T4 DNA Ligase 1 (NEB) to modified adapters (TrueQuant, GenXPro). PCR amplification was performed with KAPA HiFi Hot-Start Polymerase (KAPA Biosystems), purified by Agencourt AMPure XP beads (Beckman Coulter) and sequenced with HiSeq2000 (Illumina).

### Read alignment and transcript quantification

The 12 MACE libraries were aligned to the genome of tomato (version ITAG2.4, cv. Heinz) provided by the Sol Genomics Network (SGN; [67]). For the alignment we used NextGenMap (version 0.4.12; [68]) in single-end mode with default parameters except for the following modifications: --kmer-skip 1, −-silent-clip and --no-unal. Read alignments with an edit distance (sum of insertions, deletions and mismatches) greater than two were excluded afterwards. For quantification of transcript levels we counted reads for all genes annotated in the Generic feature format version 3 (GFF3) file of tomato with htseq-count of the High-Throughput Sequencing python framework (HTSeq; [69]). Here, we were not distinguishing between different isoforms as only a single isoform is annotated for all genes in the ITAG2.4. To account for differing sequencing depths between the MACE libraries we calculated TPM values for all genes. For this purpose, we first divided the read counts of all genes by the number of aligned reads for each library, followed by a multiplication with one million. Further, to determine if a gene was actually detected at the transcriptome level, we determined a threshold based on the established method of Loraine et al. [35] by analyzing the TPM values of genes encoding for LHC proteins (Additional file 1: Figure S1), which are not supposed to be detected due to the absence of chloroplasts in pollen. Here, we observed values up to 4.3 TPM, which led to a threshold of 5 TPM. All genes with TPM values below the threshold were subsequently set to 0 and marked as not expressed. The applied cutoff is in agreement with a previous pollen study in *A. thaliana*, where also a threshold of 5 TPM was required to identify a gene at the transcriptome level [35]. Further only genes identified in both biological replicates were determined as detected.

### Protein extraction and quantitative proteome analysis

Proteins were extracted and analyzed as previously described [70, 71]. Pollen samples were freeze-dried and ground for 2 min in a shaking mill using steel balls (2 mm diameter). The homogenized pollen sample was resuspended in 200 μL of protein extraction buffer (100 mM Tris- HCl, pH 8.0; 5% SDS, 10% glycerol; 10 mM DTT; 1% plant protease inhibitor cocktail (Sigma P9599) and incubated at room temperature for 5 min followed by incubation for 2.5 min at 95 °C and centrifugation at 21000×g for 5 min at room temperature. The supernatant was carefully transferred to a new tube. Two-hundred microliters of 1.4 M sucrose was added to the supernatant and proteins were extracted twice with 200 μL TE buffer-equilibrated phenol followed by counter extraction with 400 μL of 0.7 M sucrose. Phenol phases were combined and subsequently mixed with 2.5 volumes of 0.1 M ammonium acetate in methanol for precipitation of proteins. After 16 h of incubation at − 20 °C, samples were centrifuged for 5 min at 5000×g. The pellet was washed twice with 0.1 M ammonium acetate, once with acetone and air-dried at room temperature. The pellet was redissolved in 6 M Urea and 5% SDS and protein concentration was determined using the bicinchoninic acid assay. Proteins were prefractionated by SDS-PAGE. Forty micrograms of total protein were loaded onto a gel and run for 1.5 cm. Gels were fixed and stained with methanol: acetic acid: water: Coomassie Brilliant Blue R-250 (40:10:50:0.001). Gels were destained in methanol: water (40:60) and then each lane was divided into two fractions.

Gel pieces were destained, equilibrated and digested with trypsin, desalted and concentrated as previously described [71, 72]. Prior to mass spectrometric measurement, the tryptic peptide pellets were dissolved in 4% (*v*/v) acetonitrile, 0.1% (v/v) formic acid. 10 μg of digested peptides were injected into a one dimensional (1D) nano-flow LC-MS/MS system equipped with a pre-column (Eksigent, Germany). Peptides were eluted using an Ascentis column (Ascentis Express, peptide ES-C18 HPLC column (SUPELCO Analytical, USA), dimension 15 cm × 100 μm, pore size 2.7 μm) during a 80 min gradient from 5 to 50% (v/v) acetonitrile, 0.1% (v/v) formic acid. MS analysis was performed on an Orbitrap LTQ XL mass spectrometer (Thermo, Germany) with a controlled flow rate of 500 nL per minute. Specific tune settings for the MS were as follows: spray voltage was set to 1.8 kV; temperature of the heated transfer capillary was set to 180 °C. Each full MS scan was followed by ten MS/MS scans, in which the ten most abundant peptide molecular ions were dynamically selected, with a dynamic exclusion window set to 90 s. Ion with a + 1 or unidentified charge state in the full MS were omitted from MS/MS analysis.

### Label-free quantification of proteins

The mass spectra of the 18 LC-MS/MS libraries were screened against the tomato proteome, available from the SGN, and quantified with MaxQuant (version 1.5.4.1; [36]) using default parameters. As output MaxQuant reported LFQ intensities for all detected protein groups (proteins that share the same identified peptides), which reflect relative protein amounts comparable between the libraries analyzed in a MaxQuant run. Protein groups were expected to be present if they were identified in at least two out of three biological replicates.

### PCA and cluster analyses

The PCA was performed with the R-package FactoMineR (version 1.33; [73]) on either the TPM values (Transcriptomics) or the LFQ intensities (Proteomics) of all detected genes and protein groups, respectively. This procedure provided first insights in the developmental and conditional behavior at transcriptome and proteome level. For more detailed insights, we performed two k-means clustering approaches with the Multiple Experiment Viewer (MeV; [74]). Before clustering, we averaged TPM values and LFQ intensities of biological replicates for all detected genes and protein groups, whereby replicates with a value of zero were not taken into account. To determine the most appropriate number of clusters (k) to be produced, we created for each run a figure of merit (FOM). A FOM indicates the information gain in relation to an increasing number of clusters, whereby at some point with an additional cluster no more gain is achieved. Based on this knowledge, we determined the number of clusters leading to the last substantial gain of information and used this number for clustering. In the first clustering approach (k = 15) genes and protein groups were clustered independently based on their relative TPM values or LFQ intensities across the three developmental stages under CO. In the second clustering approach (k = 7) the genes and protein groups were clustered based on their relative TPM values and LFQ intensities across CO and HS for each developmental stage separately. Due to the fact that k-means clustering is non-deterministic, we performed for each approach (k = 15 or k = 7) 25 clusterings to subsequently determine the clustering with the lowest variance. In brief, for each clustering we calculated the averaged variance of all clusters, whereby the variance of a cluster was computed as the averaged variance of all samples in a cluster (a sample corresponds to a developmental stage or condition).

### Identification of direct and delayed translation in pollen

To analyze the behavior of genes at both transcript and protein level along the course of development, we first took the transcript and protein clusters obtained from the first clustering approach (developmental stages under CO) and created an overlap matrix, which indicated the overlap of genes between transcript and protein clusters (Additional file 1: Figure S4). For example matrix entry a_ij_ tells us the number of genes with a transcript in transcript cluster i and a protein in protein cluster j. Further, we classified subsets of the transcript and protein clusters as stage-increased (Tetrad, T; Post meiotic, PM; mature, M) if the respective stage covered two-thirds of the relative abundance in the cluster profiles. Next, we scaled the overlap of stage-increased transcript clusters (T, PM and M) with each protein cluster between 0 (minimum overlap) and 1 (maximum overlap) by linear transformation. In a similar manner we proceeded with the stage-increased protein clusters to identify transcript clusters with a high overlap. Among the overlaps with a value greater than 0.5 we observed mainly two modes of translation. The first mode implies a direct translation, at which an increase of the transcript level is directly accompanied by an increase of the protein level in the same developmental stage. Contrary, in the second mode we observed a delayed translation. Here, an increase of the transcript level and the corresponding increase of the protein level are shifted by one developmental stage. Based on this, we classified the genes as having either direct or delayed translation.

### Transcriptome and proteome changes upon HS

To compare the HS response of genes at the transcriptome and proteome level, we took the transcript and protein clusters obtained from the second clustering approach (CO and HS for each developmental stage) and calculated for each developmental stage an overlap matrix comprising genes shared between the transcript and protein clusters (shared genes). Here, clusters were ordered from a high abundance in CO to a high abundance in HS (Additional file 1: Figure S5). Next, we determined for each transcript and protein cluster the total number of shared genes. On the one hand by calculating the sum of each row (transcript clusters) and on the other hand by calculating the sum of each column (protein clusters). We then fitted on the number of shared genes distributed among the transcript and protein clusters, respectively. In addition, we performed this analysis on the complete transcript clustering without the restriction of a detected protein. The findings of this step were verified by analyzing the TPM log_2_-ratios of HS to CO (Additional file 1: Figure S6).

### Functional classification

Functional classification was carried out for genes showing direct or delayed translation in a developmental stage as well as genes up- or downregulated upon HS at the proteome level. In a first step we assigned functional categories of the KOG annotation to all annotated genes via the web server for metagenomic analysis (WebMGA; [75]) if possible. Then, we calculated for each group of genes the percentage of genes assigned to a certain functional category. In the calculation we excluded genes with no assigned category as well as those assigned to the categories R (general function prediction only) and S (function unknown) but allowed the assignment of a single gene to multiple categories by counting the gene twice or more. For simplification, in the figures only categories with more than 5% assigned genes are shown.

### Effect of HS on the Hsp and Hsf families at transcriptome and proteome level

To analyze the effect of HS on the Hsp and Hsf families we first identified all previously described family members [19] in the transcript and protein clusters of all developmental stages. In the case of protein clusters, we only considered Hsps with an identified transcript. Next, we defined the members with respect to their assignment in the HS clustering (Additional file 6: Table S5) as either downregulated (clusters Tr1 and Pr1), showing stable levels (clusters Tr2 to Tr6 and Pr2 to Pr6) or as upregulated (clusters Tr7 and Pr7). In the final step we calculated for each family the percentage of members identified with an additional distinction regarding their regulation for each developmental stage in pollen.

## Additional files

Additional file 1: **Figure S1.** Threshold estimation based on light-harvesting complex genes. **Figure S2.** Differences between developmental stages in transcriptome and proteome content. **Figure S3.** PCA plots (PC1 and 2) of pollen developmental stages under CO and HS. **Figure S4.** Overlap matrix of transcript and protein development clusters. **Figure S5.** Overlap matrix of transcript and protein HS clusters. **Figure S6.** log_2_ fold change distribution of transcript levels between HS and CO. (PDF 1405 kb)

Additional file 2: **Table S1.** Number of detected transcripts and protein groups in MACE and LC-MS/MS experiments for the biological replicates of pollen in tetrad, post-meiotic and mature stage under CO and HS. (XLSX 11 kb)

Additional file 3: **Table S2**. Transcriptomic and proteomic development clusters and genes assigned to them. (XLSX 372 kb)

Additional file 4: **Table S3.** Functional categories observed in this study. (XLSX 11 kb)

Additional file 5: **Table S4.** List of genes showing direct and delayed translation in pollen developmental stages. (XLSX 26 kb)

Additional file 6: **Table S5.** Transcriptomic and proteomic HS clusters and genes assigned to them. (XLSX 494 kb)

Additional file 7: **Table S6.** List of proteins down- and upregulated after HS in pollen developmental stages. (XLSX 37 kb)

Additional file 8: **Table S7.** List of proteins from “chaperones & protein turnover” and “translation & ribosomes” category that are differentially regulated in pollen developmental stages after HS. (XLSX 19 kb)

Additional file 9: **Table S8.** List of Hsfs and Hsps with their HS regulation at transcriptome and proteome level in pollen developmental stages. (XLSX 21 kb)

## Abbreviations

eiF: Eukaryotic translation initiation factor
HS: Heat stress
Hsf: Heat stress transcription factor
Hsp: Heat shock protein
KOG: Eukaryotic orthologous groups
LC-MS/MS: Liquid chromatography tandem-mass spectrometry
LFQ: Label-free quantification
MACE: Massive analysis of cDNA ends
PCA: Principal component analysis
RP: Ribosomal protein
TPM: Transcripts per million
