# Supplementary material for: The coupling of transcriptome and proteome adaptation during development and heat stress response of tomato pollen
Source: BMC Genomics. 2018 Jun 8;19:447. doi: 10.1186/s12864-018-4824-5 (PMC5994098; doi:10.1186/s12864-018-4824-5)
Supplement: Supplementary file 1 — Figure S1. Threshold estimation based on light-harvesting complex genes. Figure S2. Differences between developmental stages in transcriptome and proteome content. Figure S3. PCA plots (PC1 and 2) of pollen developmental stages under CO and HS. Figure S4. Overlap matrix of transcript and protein development clusters. Figure S5. Overlap matrix of transcript and protein HS clusters. Figure S6. log2 fold change distribution of transcript levels between HS and CO. (PDF 1405 kb) [file 12864_2018_4824_MOESM1_ESM.pdf]

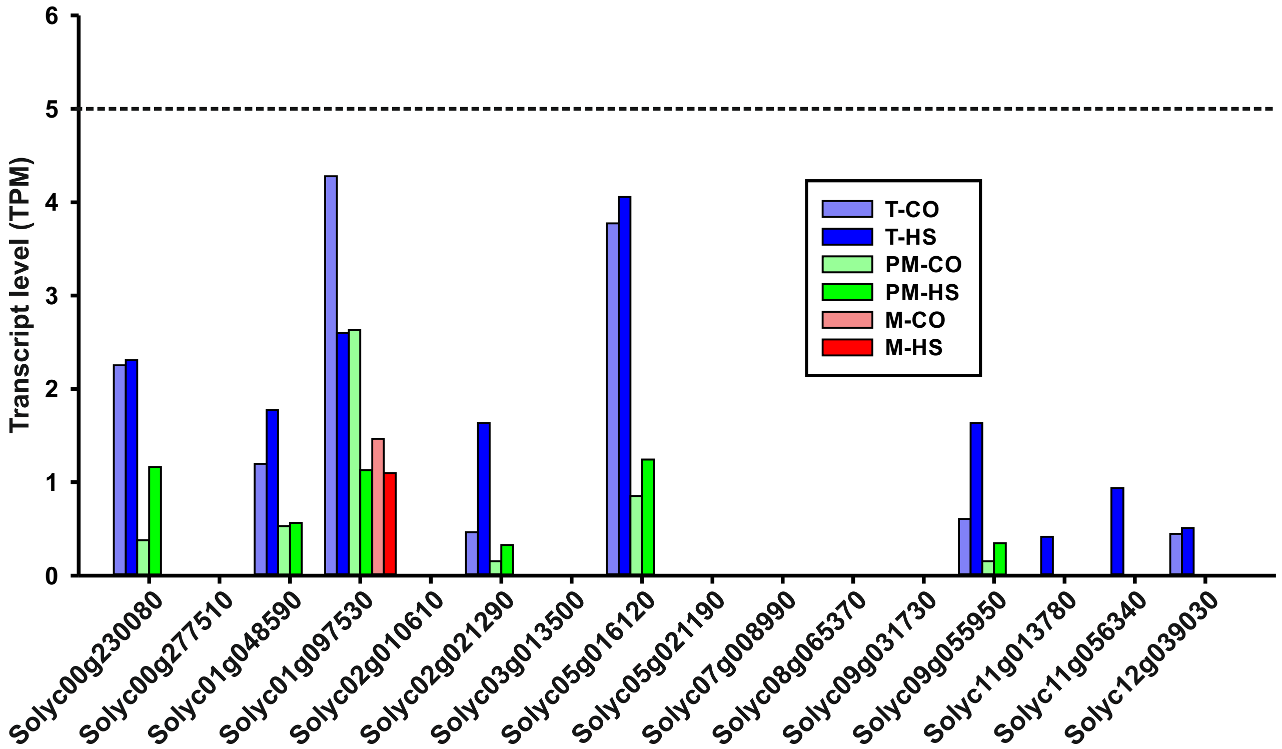

Figure S1. Threshold estimation based on light-harvesting complex genes
Shown are transcript levels (average of two biological replicates) of 16 genes encoding for light-harvesting complex proteins. Transcripts of these genes are not expected to be detected in pollen and therefore served for estimation of a suitable detection threshold. The derived threshold of 5 TPM is represented by the horizontal line.

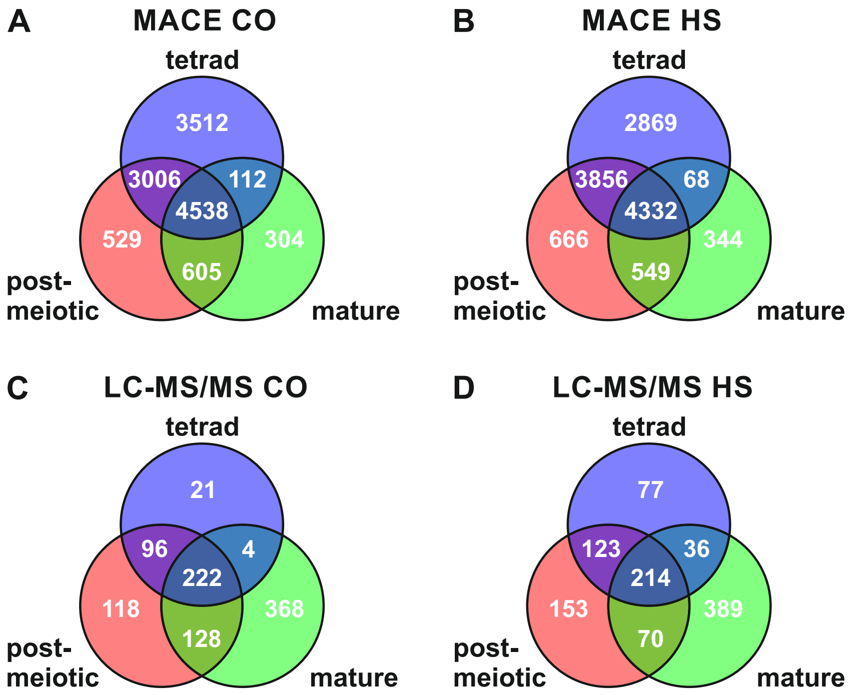

Figure S2. Differences between developmental stages in transcriptome and proteome content
Shown are venn diagrams for detected transcripts (A and B) and protein groups (C and D) under CO and HS for pollen in tetrad, post-meiotic and mature stage.

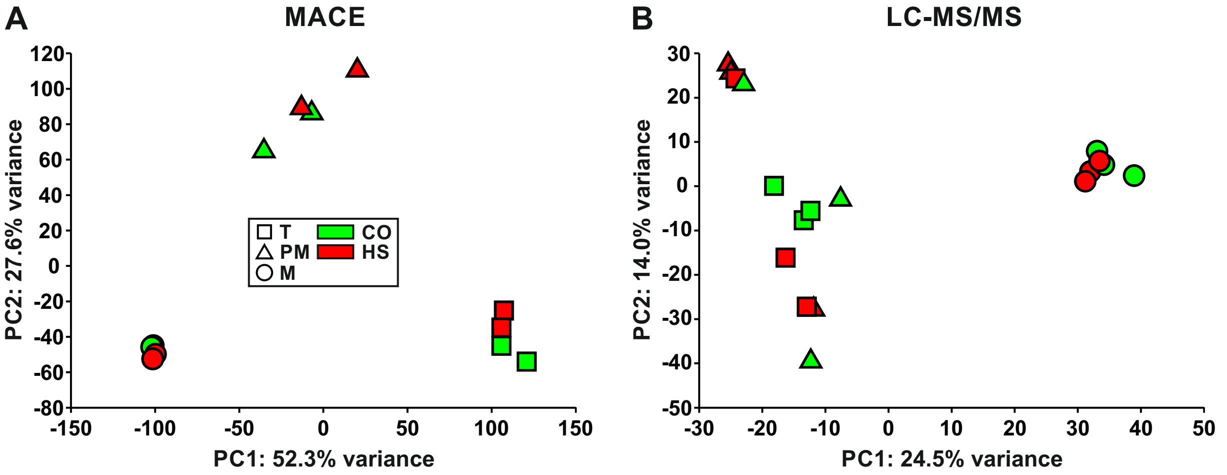

Figure S3. PCA plots (PC1 and 2) of pollen developmental stages under CO and HS
(A and B) Shown are PCA plots for tomato pollen at tetrad (square), post-meiotic (triangle) and mature stage (circle) under CO (green) and HS (red) based on measured transcript (A, MACE) and protein levels (B, LC-MS/MS). Axes are labeled with the variance explained by the first and second dimension, respectively.

		T	PM		M												
		Pr1	Pr2	Pr3	Pr4	Pr5	Pr6	Pr7	Pr8	Pr9	Pr10	Pr11	Pr12	Pr13	Pr14	Pr15	Σ_{row}
T	Tr1	13	38	0	22	2	1	31	1	3	2	32	2	1	2	0	150
	Tr2	2	15	1	14	2	0	24	0	2	1	9	5	3	1	0	79
PM	Tr3	0	1	2	13	0	1	0	2	1	0	0	0	0	0	0	20
	Tr4	0	3	0	51	13	8	0	1	1	5	0	1	1	5	0	89
M	Tr5	0	0	0	18	0	0	0	0	2	0	0	0	0	0	0	20
	Tr6	0	0	0	35	1	0	0	1	1	0	0	0	0	0	0	38
	Tr7	0	15	1	51	4	10	1	7	7	14	4	9	18	7	0	148
	Tr8	0	11	0	12	0	2	5	3	3	1	9	20	9	0	0	75
	Tr9	2	13	1	14	0	0	7	3	5	0	14	11	7	0	0	77
	Tr10	0	3	0	19	2	2	1	1	1	1	1	6	1	0	0	38
	Tr11	2	1	0	48	7	7	1	1	1	1	1	3	4	5	1	83
	Tr12	2	16	1	45	3	6	5	4	10	7	5	30	21	7	1	163
	Tr13	0	0	0	8	2	0	0	0	0	0	0	1	0	1	0	12
	Tr14	2	15	3	24	0	0	7	4	2	2	7	10	4	1	0	81
	Tr15	2	1	0	69	4	0	0	1	1	2	0	1	1	1	2	85
	Σ_{column}	25	132	9	443	40	37	82	29	40	36	82	99	70	30	4	

Figure S4. Overlap matrix of transcript and protein development clusters
Shown is the overlap of genes between transcript (Tr) and protein (Pr) clusters. Genes were clustered based on their relative transcript or protein levels across tetrads, post-meiotic and mature pollen. Next to each cluster the corresponding profile is given. In addition, tetrad (T), post-meiotic (PM) and mature (M) increased transcript and protein clusters were defined.

A

	Pr1	Pr2	Pr3	Pr4	Pr5	Pr6	Pr7	Σ_{row}
Tr1	2	0	1	1	4	0	4	12
Tr2	5	11	11	14	13	0	13	67
Tr3	10	16	30	44	11	6	38	155
Tr4	14	18	44	35	14	3	55	183
Tr5	13	17	25	25	9	2	33	124
Tr6	3	3	3	15	4	4	5	37
Tr7	0	1	1	2	0	1	11	16
Σ_{column}	47	66	115	136	55	16	159	

B

	Pr1	Pr2	Pr3	Pr4	Pr5	Pr6	Pr7	Σ_{row}
Tr1	1	0	2	1	0	2	1	7
Tr2	9	6	3	7	6	6	7	44
Tr3	32	12	17	15	29	13	21	139
Tr4	61	11	36	55	69	17	44	293
Tr5	41	4	18	38	41	15	40	197
Tr6	10	5	9	8	9	1	4	46
Tr7	5	1	5	6	5	1	17	40
Σ_{column}	159	39	90	130	159	55	134	

C

	Pr1	Pr2	Pr3	Pr4	Pr5	Pr6	Pr7	Σ_{row}
Tr1	3	0	0	8	7	2	2	22
Tr2	6	3	20	53	48	14	4	148
Tr3	28	9	46	92	80	35	33	323
Tr4	32	9	41	68	74	24	23	271
Tr5	6	1	7	10	20	4	4	52
Tr6	1	0	1	2	2	2	0	8
Tr7	2	0	0	2	1	0	3	8
Σ_{column}	78	22	115	235	232	81	69	

Figure S5. Overlap matrix of transcript and protein HS clusters
Shown is the overlap of genes between transcript (Tr) and protein (Pr) clusters for tetrads (A), post-meiotic (B) and mature pollen (C). Genes were clustered based on their relative transcript or protein levels across CO and HS. Next to each cluster the corresponding profile is given.

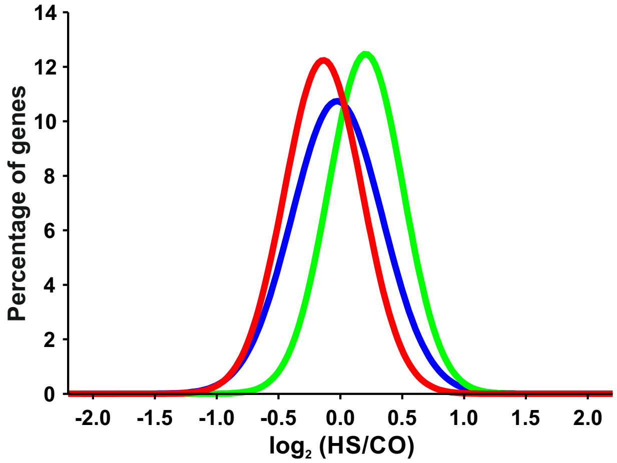

Figure S6. $\log_2$ fold change distribution of transcript levels between HS and CO
Shown is the $\log_2$ fold change distribution of transcript levels (TPM) between HS and CO for tetrads (blue), post-meiotic (green) and mature pollen (red).
